# Estimating optimal individualized treatment rules with multistate processes

**DOI:** 10.1111/biom.13864

**Published:** 2023-04-17

**Authors:** Giorgos Bakoyannis

**Affiliations:** Department of Biostatistics and Health Data Science, Indiana University, Indianapolis, Indiana, USA

**Keywords:** multistate model, outcome-weighted learning, patient preferences, precision medicine, response

## Abstract

Multistate process data are common in studies of chronic diseases such as cancer. These data are ideal for precision medicine purposes as they can be leveraged to improve more refined health outcomes, compared to standard survival outcomes, as well as incorporate patient preferences regarding quantity versus quality of life. However, there are currently no methods for the estimation of optimal individualized treatment rules with such data. In this paper, we propose a nonparametric outcome weighted learning approach for this problem in randomized clinical trial settings. The theoretical properties of the proposed methods, including Fisher consistency and asymptotic normality of the estimated expected outcome under the estimated optimal individualized treatment rule, are rigorously established. A consistent closed-form variance estimator is provided and methodology for the calculation of simultaneous confidence intervals is proposed. Simulation studies show that the proposed methodology and inference procedures work well even with small-sample sizes and high rates of right censoring. The methodology is illustrated using data from a randomized clinical trial on the treatment of metastatic squamous-cell carcinoma of the head and neck.

## 1 | INTRODUCTION

Modern precision medicine acknowledges the heterogeneity of the majority of human diseases and aims to develop and deliver therapies that are tailored to the individual patient. At the heart of these efforts is the development of data-driven individualized treatment assignment rules (Kosorok & Laber, 2019). The purpose of such rules is to provide the right treatment to a given patient (Lipkovich et al., 2017) and, thereby, to improve health outcomes among patients overall. Over the past decade, there has been a large number of new statistical methods for the estimation of optimal individualized treatment rules (ITRs) with various types of outcomes, including continuous, binary, survival, and competing risks outcomes. However, to the best of our knowledge, there are currently no methods for the estimation of optimal ITRs with stochastic processes that evolve through multiple discrete states over (continuous) time, also known as *multistate processes*. Such processes are commonly encountered in studies of chronic diseases, such as cancer and HIV infection, where disease evolution is often characterized by multiple discrete health states. An example is the SPECTRUM trial (Vermorken et al., 2013), a phase III randomized clinical trial on recurrent or metastatic squamous-cell carcinoma of the head and neck, where patient event history was characterized by the states “tumor response” (i.e., tumor shrinkage per Therasse et al. 2000), “disease progression”, and “death.” This paper addresses the issue of optimal ITR estimation with right-censored multistate processes data in randomized clinical trial settings.

Multistate process data are ideal for precision medicine purposes as they can be leveraged to improve more refined health outcomes (compared to, e.g., overall or progression-free survival) as well as incorporate patient preferences. Typically, such data contain information about one or more transient health states (e.g., tumor response), that is not fully captured by standard survival or competing risks data. Therefore, they provide more comprehensive information about more refined health outcomes that may reflect both quantity and quality of life. Improvement of such outcomes may be more desirable than mere life extension to both patients and clinicians. In oncology, for example, such a desirable health outcome is sustained tumor response, which is associated with better quality of life, extended progression-free survival (PFS) time, and a prolonged treatment-free interval (Kaufman et al., 2017). Tumor response was also a desirable outcome in the SPECTRUM trial. Another important outcome is extended quality-adjusted lifetime, which can be defined as the weighted sum of the times spent in a set of desirable health states (Oza et al., 2020; Pelzer et al., 2017). Quality-adjusted lifetime can be defined in multiple different ways according to patient preferences by using different weighting schemes that reflect the needs of different groups of patients. In this way, individual priorities toward quality versus quantity of life can be taken into account.

The methods for optimal ITR estimation can be classified into two broad categories: (i) backward induction methods such as Q-learning (Murphy, 2005; Zhao et al., 2009) and A-learning (Murphy, 2003; Robins, 2004) and (ii) direct-search methods, also known as policy-search methods, such as outcome weighted learning (Zhao et al., 2012) and value search estimation (Zhang et al., 2012). The first class of methods typically estimates the optimal ITR by modeling either the conditional expectation of the outcome given the covariates (Q-learning) or the interactions between treatment and the covariates (A-learning). The second class of methods estimates the optimal ITR directly by optimizing an appropriate objective function. Given that imposing a realistic model for the conditional mean outcome or the treatment interactions is difficult for general multistate processes, the focus in this paper is on direct-search methods. The issue of ITR estimation with survival outcomes has been well addressed in the literature. Zhao et al. (2015) extended the outcome-weighted learning framework of Zhao et al. (2012) to situations, where the outcome of interest is a right-censored survival time by proposing (i) an inverse censoring weighting (ICO) and (ii) a doubly robust (DR) outcome-weighted learning approach. Estimation in these approaches relies on a weighted version of support vector machines (Cortes & Vapnik, 1995). A similar method for ITR estimation with survival outcomes was proposed by Bai et al. (2017). A value search estimation approach for maximizing the t-year survival probability was developed by Jiang et al. (2017). This method approximates the nonsmooth objective function of the problem by a smooth one using kernel smoothing. Recently, there has also been some interest in the issue of ITR estimation with competing risk outcomes (He et al., 2021; Zhou et al., 2021). Nevertheless, the methods for survival and competing risk outcomes cannot be used for the estimation of optimal ITRs with right-censored multistate processes.

In this work, we extend the outcome-weighted learning framework (Zhao et al., 2012, 2015) to deal with situations, where the outcome of interest is an arbitrary right-censored multistate process, incorporating also patient preferences. The proposed method does not impose Markov assumptions or model assumptions on the multistate process of interest. The novelty of this paper is twofold. First, we devise a novel objective function which, in contrast to the ICO approach by Zhao et al. (2015), utilizes information from the censored cases. Importantly, this is achieved without imposing and estimating a model for the conditional expectation of the outcome given treatment and the covariates, as opposed to the DR approach by Zhao et al. (2015). Second, in addition to showing Fisher and universal consistency of the proposed method, we establish the asymptotic distribution of the proposed estimator for the expected outcome under the estimated optimal ITR and derive a consistent closed-form variance estimator. Based on our theoretical results, we also propose a method for the calculation of simultaneous confidence intervals over a set of patient preferences to account for multiplicity. The simulation studies provide evidence that the proposed estimator and inference procedures work well even with small-sample sizes and under high rates of right censoring. Furthermore, the simulation studies reveal that the proposed method performs better than the previously proposed ICO and DR approaches for censored failure times.

## 2 | METHODOLOGY

### 2.1 | Notation and data

Consider a multistate process {X(t):t∈[0,τ]} with (finite) state space S={1,…,S}, where τ<∞ is the maximum observation time. Let $Z\in 𝒵\subset R^{p}$ denote a vector of baseline covariates that may be related to the effect of treatment on the multistate process of interest. For simplicity, the treatment A is considered to be a binary variable taking its values in the treatment set {-1,1}. With multistate processes, the goal of treatment is typically to prolong the time spent in a set of desirable health states. Given that some health states may be more desirable than others and that patient preferences may vary, we define the benefit processes

$$\{Y_{w}(t)=w^{\prime }\tilde{X}(t)\;:t\in [0,\;\tau ],\;w\in 𝒲\},$$

where $\tilde{X}(t)=(I\{X(t)=1\},\ldots ,I\{X(t)=S\}{)}^{\prime },\;w$ is an S-dimensional vector of preference weights that satisfies 0≤w≤1 and the sum of its elements is positive and less than S, and $𝒲=\left\{w_{1},\ldots ,w_{M}\right\}$ is a prespecified finite set of preference weight vectors that reflect different patient preferences/priorities. Based on the latter processes, we define the utilities

(1) $$\int_{0}^{\tau }\;Y_{w}(t)dm(t),\;w\in 𝒲,$$

where the integrator function is m(t)=t and induces the Lebesgue measure on the Borel σ-algebra ℬ([0,τ]). Since $\int_{0}^{\tau }\;I\{X(t)=j\}dm(t)$ is the time spent in the jth state during the time interval [0,τ], the utilities (1) represent weighted sums of the (restricted) times spent in each state. For example, consider the states “initial disease state” (state 1), “tumor response” (state 2), and “disease progression or death” (state 3), in the setting of the SPECTRUM trial mentioned in Section 1. A potential choice for the set of preference weights in this example is $𝒲=\left\{(0,\;1,\;0{)}^{\prime },\;(0.5,\;1,\;0{)}^{\prime },\;(1,\;1,\;0{)}^{\prime }\right\}$. When $w=(0,\;1,\;0{)}^{\prime }$, the utility $\int_{0}^{\tau }\;Y_{w}(t)dm(t)=\int_{0}^{\tau }\;I\{X(t)=2\}dm(t)$ corresponds to the restricted time spent in the tumor response state. The utility under the choice $w=(0.5,\;1,\;0{)}^{\prime }$ corresponds to the restricted quality-adjusted lifetime $\int_{0}^{\tau }\;Y_{w}(t)dm(t)=0.5\times \int_{0}^{\tau }\;I\{X(t)=1\}dm(t)+\int_{0}^{\tau }\;I\{X(t)=2\}dm(t)$. This utility represents the restricted (quality-adjusted) time lived, where the time spent in the initial disease state (i.e., without tumor response) is reduced by 50% to reflect a quality of life loss due to disease symptoms and/or side effects due to treatment continuation. When $w=(1,\;1,\;0{)}^{\prime }$, the utility is the (restricted) PFS time, in which the times lived in the initial disease state and the tumor response state are equally important. Different choices of w reflect different patient priorities towards quality versus quantity of life.

In practice, the multistate and benefit processes are not fully observed for all individuals due to the usual right censoring. Let C denote the right censoring time and $T^{*}$ the time of arrival at an absorbing state (i.e., death). Letting $T=T^{*}\wedge \tau$, with a∧b=min(a,b) for any $a,\;b\in R,\;{\tilde{T}}_{i}=T_{i}\wedge C_{i}$, and $\Delta_{i}=I\left(T_{i}\leq C_{i}\right)$, the observed data consist of independent and identically distributed copies of $D_{i}=\left(Z_{i},A_{i},{\tilde{T}}_{i},\Delta_{i},\left\{X_{i}(t)I\left(C_{i}\geq T_{i}\wedge t\right)\;:t\in [0,\tau ]\right\}\right),\;i=1,\ldots ,n$, where $X_{i}(t)I\left(C_{i}\geq T_{i}\wedge t\right),\;t\in [0,\tau ]$, is the censored version of the multistate process which is equal to 0 for the censored individuals after their censoring time. Note that $Y_{i,w}(t)=w^{\prime }{\left(I\left\{X_{i}(t)=1\right\},\ldots ,I\left\{X_{i}(t)=S\right\}\right)}^{\prime }$ is the benefit process for the ith individual.

### 2.2 | Optimal individualized treatment rule estimation with multistate processes

An ITR is a deterministic function d:𝒵↦{-1,1} which suggests treatment choice d(z)∈{-1,1} for a patient with covariates Z=z. Since the estimation of an optimal ITR is essentially a causal inference problem, we utilize the potential outcomes causal framework (Rubin, 2005; Tsiatis et al., 2019). Let $Y_{w}^{*}(t;1)$ and $Y_{w}^{*}(t;-1)$, w∈𝒲, denote the potential benefit processes under treatment choices 1 and −1, respectively, at time t∈[0,τ]. Since the potential outcomes cannot be directly observed in real-world settings, we need to impose the following causal assumptions.

A1. Stable unit treatment value assumption: $Y_{w}(t)=Y_{w}^{*}(t;1)I(A=1)+Y_{w}^{*}(t;-1)I(A=-1)$, t∈[0,τ].

A2. Independent treatment assignment assumption: $\left\{Y_{w}^{*}(t;1),Y_{w}^{*}(t;-1),Z\}⫫A,\;t\in [0,\tau \right]$.

A3. Positivity assumption: $\pi_{0}=P(A=1)\in \left[c_{1},c_{2}\right]$, with $0<c_{1}<c_{2}<1$.

Assumptions A2 and A3 are automatically satisfied in randomized clinical trials. It must be highlighted that assumption A1 implies that the *observed* benefit process $\left\{Y_{w}(t)\;:t\in [0,\tau ]\right\}$, unlike the *potential* benefit processes $\left\{Y_{w}^{*}(t;1),Y_{w}^{*}(t;-1)\;:t\in [0,\tau ]\right\}$, is associated with treatment A. Now, we can define the potential benefit processes under an ITR d as

$$\begin{array}{l} Y_{w}^{*}(t;d)=Y_{w}^{*}\left(t;1\right)I\left\{d\left(Z\right)=1\right\}+Y_{w}^{*}\left(t;-1\right)I\left\{d\left(Z\right)=-1\right\},\\ \;w\in 𝒲,t\in [0,\tau ]. \end{array}$$

These processes are essential for defining potential benefit under an ITR for multistate processes. For such processes, we define the *value* functions as

$${𝒱}_{w}(d)=E\left\{\int_{0}^{\tau }\;Y_{w}^{*}(t;d)dm(t)\right\},w\in 𝒲,$$

where ${𝒱}_{w}(d)$ is the expected sum of the weighted (under w) times spent in each state of the process during the time interval [0,τ], under the ITR d. Under assumptions A1–A3, the independent right censoring assumption, and the fact that

$$\frac{Y_{w}(t)I(C\;\geq \;T\;\wedge \;t)}{exp\left\{-\Lambda_{0}(T\;\wedge \;t)\right\}}=\frac{Y_{w}(t)I(C\;\geq \;T\;\wedge \;t)}{exp\{-\Lambda_{0}(\tilde{T}\;\wedge \;t)\}},\;t\in \left[0,\tau \right],w\in 𝒲,$$

it can be shown that the value functions can be expressed in terms of the observable data as

$${𝒱}_{w}(d)=E\left(\left[\int_{0}^{\tau }\frac{Y_{w}(t)I(C\;\geq \;T\;\wedge \;t)}{\exp\left\{-\Lambda_{0}(\tilde{T}\;\wedge \;t)\right\}}\text{d}m(t)\right]\frac{I(A\;=\;d(Z))}{A\pi_{0}\;+\;(1\;-\;A)/2}\right),\;w\in 𝒲,$$

where $\Lambda_{0}(t)$ is the cumulative hazard function of the right censoring variable C at time t. An optimal ITR $d_{w}^{*},\;w\in 𝒲$, is a maximizer of the corresponding value function, that is,

$$d_{w}^{*}\in \underset{d}{\arg max}{𝒱}_{w}\left(d\right).$$

Given that any rule d:𝒵↦{-1,1} can be expressed as d(z)=sgn⁡{f(z)}, where sgn⁡(x)=I(x≥0)-I(x<0), for some measurable function f:𝒵↦R, and since

$$\begin{array}{l} {𝒱}_{w}(d)=E\left(\left[\int_{0}^{\tau }\;\frac{Y_{w}(t)I(C\;\geq \;T\;\wedge \;t)}{exp\{-\Lambda_{0}(\tilde{T}\;\wedge \;t)\}}dm(t)\right]\frac{1}{A\pi_{0}\;+\;(1\;-\;A)/2}\right)\\ \;-E\left(\left[\int_{0}^{\tau }\;\frac{Y_{w}(t)I(C\;\geq \;T\;\wedge \;t)}{exp\{-\Lambda_{0}(\tilde{T}\;\wedge \;t)\}}dm(t)\right]\frac{I(A\;\neq \;d(Z))}{A\pi_{0}\;+\;(1\;-\;A)/2}\right), \end{array}$$

an optimal ITR is $d_{w}^{*}(z)=sgn\{f_{w}^{*}(z)\}$, where $f_{w}^{*}$ is a minimizer of the risk function

$$\begin{array}{l} {ℛ}_{w}(f)\;=E\left(\left[\int_{0}^{\tau }\;\frac{Y_{w}(t)I(C\;\geq \;T\;\wedge \;t)}{exp\{-\Lambda_{0}(\tilde{T}\;\wedge \;t)\}}dm(t)\right]\frac{I(A\;\neq \;sgn(f(Z))}{A\pi_{0}\;+\;(1\;-\;A)/2}\right)\\ \;=E\left(\left[\int_{0}^{\tau }\;\frac{Y_{w}(t)I(C\;\geq \;T\;\wedge \;t)}{exp\{-\Lambda_{0}(\tilde{T}\;\wedge \;t)\}}dm(t)\right]\frac{I(Af(Z)\;<\;0)}{A\pi_{0}\;+\;(1\;-\;A)/2}\right), \end{array}$$

over all measurable functions f:𝒵↦R. It is not hard to see that

$${𝒱}_{w}(sgn(f_{w}^{*}))-{𝒱}_{w}(sgn(f))={ℛ}_{w}(f)-{ℛ}_{w}(f_{w}^{*}),\;w\in 𝒲,$$

for any measurable f. Minimizing ${ℛ}_{w}(f)$ over all measurable functions is clearly not feasible. Therefore, we will consider minimization over a subset of the class of all measurable functions f:𝒵↦R. In particular, we consider either of the following subsets.

- Class of linear functions $\left\{f(\cdot )=\beta_{0}+\left\langle \beta_{1},\;\cdot \right\rangle \;:\beta_{0}\in \right.\left.R,\beta_{1}\in R^{p}\right\}$, where $\left\langle \beta_{1},\;z\right\rangle =\beta_{1}^{\prime }z$.
- Reproducing kernel Hilbert space (RKHS) ${ℋ}_{k}$ with kernel k, which is the completion of the space $\left\{f(\cdot )=\{\sum_{j=1}^{m}\;\alpha_{j}k\left(\cdot ,z_{j}\right)\;:m\in N,z_{j}\in 𝒵,\alpha_{j}\in R\right\}$. Here, we consider the RKHS with the Gaussian kernel, $k\left(z_{1},z_{2}\right)=exp(-\sigma_{n}{∥z_{1}-z_{2}∥}^{2}),\;z_{1},\;z_{2}\in 𝒵$.

Minimizing the empirical version of ${ℛ}_{w}(f)$, over the chosen class ℱ, is challenging because it involves a discontinuous and nonconvex function of f. To alleviate, we follow the paradigm of outcome weighting learning (Zhao et al., 2012, 2015) and support vector machines (Steinwart & Christmann, 2008) and utilize the surrogate risk

$${ℛ}_{\phi ,w}(f)=E\left(\left[\int_{0}^{\tau }\;\frac{Y_{w}(t)I(C\;\geq \;T\;\wedge \;t)}{exp\{-\Lambda_{0}(\tilde{T}\;\wedge \;t)\}}dm(t)\right]\frac{\phi (Af(Z))}{A\pi_{0}\;+\;(1\;-\;A)/2}\right),$$

where ϕ(x)=max(0,1-x) is the hinge loss, which is convex in f. The cumulative hazard function $\Lambda_{0}$ of the right censoring distribution can be estimated using the nonparametric Nelson–Aalen estimator ${\overset{ˆ}{\Lambda }}_{n}(t)=\sum_{i=1}^{n}\;\int_{0}^{t}\;{\left\{\sum_{i=1}^{n}\;Y_{i}(u)\right\}}^{-1}dN_{i}(u)$, t∈[0,τ], where $N_{i}(t)=\left(1-\Delta_{i}\right)I\left({\tilde{T}}_{i}\leq t\right)$ and $Y_{i}(t)=I\left({\tilde{T}}_{i}\geq t\right)$. An obvious estimator of $\pi_{0}$ is ${\overset{ˆ}{\pi }}_{n}=n^{-1}\sum_{i=1}^{n}\;I\left(A_{i}=1\right)$. Thus, the empirical version of the surrogate risk ${ℛ}_{\phi ,w}$ is

(2) $${\hat{ℛ}}_{\phi ,w}\left(f\right)=\frac{1}{n}\sum_{i=1}^{n}\left(\left[\int_{0}^{\tau }\frac{Y_{i,w}\left(t\right)I\left(C_{i}\;\geq \;T_{i}\;\wedge \;t\right)}{\text{exp}\left\{-{\hat{\text{Λ}}}_{n}\left({\tilde{T}}_{i}\;\wedge \;t\right)\right\}}dm\left(t\right)\right]\frac{\phi \left(A_{i}f\left(Z_{i}\right)\right)}{A_{i}{\hat{\pi }}_{n}\;+\;\left(1\;-\;A_{i}\right)/2}\right).$$

Note that, even though $\pi_{0}$ is known in clinical trials, the estimate ${\overset{ˆ}{\pi }}_{n}$ is used in Equation (2) as this typically leads to some efficiency gain in inverse probability weighting type estimators (Tsiatis et al., 2019). The empirical surrogate risk for the ICO estimator for censored failure times (e.g., $\tilde{T}$) by Zhao et al. (2015) for the setting considered here is

$$\frac{1}{n}\sum_{i=1}^{n}\left(\left[\frac{\Delta_{i}{\tilde{T}}_{i}}{exp\{-{\overset{ˆ}{\Lambda }}_{n}({\tilde{T}}_{i})\}}\right]\frac{\phi \left(A_{i}f\left(Z_{i}\right)\right)}{A_{i}\pi_{0}\;+\;\left(1\;-\;A_{i}\right)/2}\right).$$

The latter incorporates inverse censoring weighting in the censored time of interest and discards the censored observations $\left(\Delta_{i}=0\right)$. In contrast, the proposed empirical surrogate risk (2) utilizes information from both uncensored and censored cases by incorporating inverse censoring weighting in the underlying stochastic process $\left\{Y_{w}(t)\;:t\in [0,\tau ]\right\}$. Importantly, this is achieved without imposing and estimating a model for the conditional expectation of the time of interest given A and Z, as opposed to the DR approach (Zhao et al., 2015). Simulation studies summarized in Section 4 reveal that these characteristics of the proposed method lead to a better performance compared to the ICO and DR estimators (Zhao et al., 2015) for censored failure times.

Similarly to Zhao et al. (2012, 2015), the estimators of the optimal decision functions within the class ℱ are obtained using penalized minimization of ${\hat{ℛ}}_{\phi ,w}$ as

$${\overset{ˆ}{f}}_{n,w,\lambda_{n}}=\underset{f\in ℱ}{argmin}\{{\hat{ℛ}}_{\phi ,w}(f)+\lambda_{n}∥f{∥}^{2}\},\;w\in 𝒲,$$

where $\lambda_{n}$ is a positive tuning parameter that controls the complexity of f and ∥⋅∥ is a norm on the chosen class ℱ. For example, ∥⋅∥ is the Euclidean norm if ℱ is the class of linear functions. For notational simplicity, we omit the subscript $\lambda_{n}$ from the estimated decision functions and use the more compact notation ${\overset{ˆ}{f}}_{n,w}$. Based on the estimated decision functions $\left\{{\overset{ˆ}{f}}_{n,w}\;:w\in 𝒲\right\}$ the estimated optimal ITRs are

$${\overset{ˆ}{d}}_{n,w}(z)=sgn\{{\overset{ˆ}{f}}_{n,w}(z)\},\;w\in 𝒲,\;z\in 𝒵.$$

Based on the class of estimated ITRs $\left\{{\overset{ˆ}{d}}_{n,w}\;:w\in 𝒲\right\}$, treatment assignment for a given patient utilizes ${\overset{ˆ}{d}}_{n,w}$, for the weight vector w that is closest, with respect to the Euclidean norm, to the preference weight $w_{0}$ that reflects the patient’s own preferences/priorities.

### 2.3 | Estimation of the value function

The value function of an ITR d can be estimated as

$$\begin{array}{l} {\hat{𝒱}}_{n,w}(d)=\frac{1}{n}\sum_{i=1}^{n}\;\left(\left[\int_{0}^{\tau }\;\frac{Y_{i,w}(t)I\left(C_{i}\;\geq \;T_{i}\;\wedge \;t\right)}{exp\{-{\overset{ˆ}{\Lambda }}_{n}({\tilde{T}}_{i}\;\wedge \;t)\}}dm(t)\right]\right.\\ \;\left.\times \frac{I\left(A_{i}\;=\;d\left(Z_{i}\right)\right)}{A_{i}{\overset{ˆ}{\pi }}_{n}\;+\;\left(1\;-\;A_{i}\right)/2}\right),\;w\in 𝒲. \end{array}$$

The value ${\hat{𝒱}}_{n,w}(d)$ can be seen as the (estimated) performance of the ITR d under the preference weight vector w. The estimated value of the estimated optimal ITR ${\hat{𝒱}}_{n,w}({\overset{ˆ}{d}}_{n,w})$ is expected to be an over-optimistic estimate of the performance of ${\overset{ˆ}{d}}_{n,w}$ in a future (out of sample) patient when sample size is small relatively to p or relatively to the complexity of ℱ. This is because the estimator ${\hat{𝒱}}_{n,w}({\overset{ˆ}{d}}_{n,w})$ uses the same set of data $\left\{D_{i}\;:i=1,\ldots ,n\right\}$, for both the estimation of the optimal ITR and the evaluation of its performance. This phenomenon is similar to the behavior of the training error in support vector machines (Hastie et al., 2009). A better estimate of ${𝒱}_{w}({\overset{ˆ}{d}}_{n,w})$ in finite samples is expected to be the jackknife or leave-one-out cross-validation value estimator

(3) $$\begin{array}{l} {\hat{𝒱}}_{n,w}^{jk}\left({\hat{d}}_{n,w}\right)=\frac{1}{n}\sum_{i=1}^{n}(\left[\int_{0}^{\tau }\frac{Y_{i,w}(t)I\left(C_{i}\;\geq \;T_{i}\;\wedge \;t\right)}{\exp\left\{-{\hat{\Lambda }}_{n}\left({\tilde{T}}_{i}\;\wedge \;t\right)\right\}}dm(t)\right]\\ \;\times \frac{I\left(A_{i}\;=\;{\hat{d}}_{n,w}^{\left(-i\right)}\left(Z_{i}\right)\right)}{A_{i}{\hat{\pi }}_{n}\;+\;\left(1\;-\;A_{i}\right)/2}),\;w\in 𝒲, \end{array}$$

where ${\overset{ˆ}{d}}_{n,w}^{\left(-i\right)}=sgn({\overset{ˆ}{f}}_{n,w}^{\left(-i\right)})$ is the optimal ITR estimated under the preference weight vector w using all but the data of the ith individual.

## 3 | THEORETICAL PROPERTIES

The first theorem justifies the use of the surrogate risk ${ℛ}_{\phi ,w}$, instead of the original risk ${ℛ}_{w}$, for the estimation of optimal ITR $d_{w}^{*},\;w\in 𝒲$.

### Theorem 1 (Fisher consistency).

Suppose that assumptions A1–A3 and condition C1 in Web Appendix A hold. Then, for any w∈𝒲, if ${\tilde{f}}_{w}$ minimizes ${ℛ}_{\phi ,w},\;d_{w}^{*}(z)=sgn\left\{{\tilde{f}}_{w}(z)\right\}$ for all z∈𝒵.

The proof of Theorem 1 can be found in Web Appendix A.1. The next theorem ensures that ${ℛ}_{\phi ,w}({\overset{ˆ}{f}}_{n,w})$, which is the true surrogate risk of the estimated decision function ${\overset{ˆ}{f}}_{n,w}$, converges (in probability) to the minimal surrogate risk over the chosen class ℱ. It also asserts that, if the chosen class ℱ is appropriate, then the proposed estimator ${\overset{ˆ}{d}}_{n,w}$ is universally consistent, that is, its value converges (in probability) to the optimal value ${𝒱}_{w}(d_{w}^{*})$.

### Theorem 2.

Suppose that assumptions A1–A3 and conditions C1, C3, and C4 in Web Appendix A hold. Then, for $\lambda_{n}>0$ with $\lambda_{n}\to 0$, and $n\lambda_{n}\to \infty$,

$$\left|{ℛ}_{\phi ,w}({\overset{ˆ}{f}}_{n,w})-\underset{f\in ℱ}{inf}\;{ℛ}_{\phi ,w}(f)\right|\overset{p}{\to }0,\;w\in 𝒲,$$

as n→∞, for any distribution P of the data D. Moreover, if (i) ℱ is the space of linear functions and $f_{w}^{*}\in ℱ$ or (ii) ℱ is the RKHS with the Gaussian kernel and the marginal distribution μ of Z is regular, then

$$\left|{𝒱}_{w}({\overset{ˆ}{d}}_{n,w})-{𝒱}_{w}(d_{w}^{*})\right|\overset{p}{\to }0,\;\text{as}\;n\to \infty .$$

Theorem 2 is proved in Web Appendix A.2. When ℱ is the space of linear functions, universal consistency requires that the optimal decision function $f_{w}^{*}$ is linear, that is, $f_{w}^{*}\in ℱ$. This requirement can be made more plausible by considering an enlarged covariate space $\tilde{𝒵}$ that includes polynomial terms and/or two-way interaction terms between the original covariates Z. If $f_{w}^{*}\notin ℱ,\;{𝒱}_{w}({\overset{ˆ}{d}}_{n,w})$ is expected to converge to a value that is lower than the optimal value ${𝒱}_{w}(d_{w}^{*})$. Nevertheless, the limit of ${𝒱}_{w}({\overset{ˆ}{d}}_{n,w})$ can be seen as an approximation to the optimal value ${𝒱}_{w}(d_{w}^{*})$ by the first statement of Theorem 2 and the fact that ${ℛ}_{w}(f_{w}^{*})\leq {ℛ}_{w}(f)\leq {ℛ}_{\phi ,w}(f)$ for any f∈ℱ, since the hinge loss satisfies ϕ(x)≥I(x<0) for all x∈R.

Interestingly, when ℱ is the RKHS with the Gaussian kernel and the marginal distribution of Z is regular, the estimated ITR is always universally consistent. However, the so-called no-free-lunch theorem (Steinwart & Christmann, 2008) implies that the corresponding rate of convergence can be extremely slow for at least some distributions of the data. This means that an extremely large sample size may be required in practice in order to obtain an ITR ${\overset{ˆ}{d}}_{n,w}$ with a value reasonably close to the optimal value. For this reason, we will restrict our attention to the case where ℱ is the space of linear functions in the remainder of this paper.

The next theorem characterizes the asymptotic distribution of the estimated value function of a fixed decision function f.

### Theorem 3.

Under assumptions A1–A3 and conditions C1, C2, and C4 in Web Appendix A, we have that

$$\begin{array}{l} G_{n,w}(f)\;:=\sqrt{n}\{{\hat{𝒱}}_{n,w}(sgn(f))-{𝒱}_{w}(sgn(f))\}\\ \;=\frac{1}{\sqrt{n}}\sum_{i=1}^{n}\;\psi_{i,w}(f)+\epsilon_{n,w}(f),\;w\in 𝒲, \end{array}$$

for any given measurable decision function f, where the explicit formula for the influence function $\psi_{i,w}(f)$ is provided in Web Appendix B and $\epsilon_{n,w}(f)=o_{p}(1)$. Moreover, if ℱ is the space of linear functions, then ${sup}_{f\in ℱ}\;\left|\epsilon_{n,w}(f)\right|=o_{p}(1)$ and the class of influence functions $\left\{\psi_{w}(f)\;:f\in ℱ\right\}$ is P-Donsker.

The proof of Theorem 3 is given in Web Appendix A.3. This theorem implies that, for any measurable decision function $f,\;G_{n,w}(f)$ is asymptotically normal with mean zero and variance $\sigma_{w}^{2}(f)=E\psi_{1,w}^{2}(f)$. This asymptotic variance can be consistently (in probability) estimated by ${\overset{ˆ}{\sigma }}_{w}^{2}(f)=n^{-1}\sum_{i=1}^{n}\;{\overset{ˆ}{\psi }}_{i,w}^{2}(f)$, where ${\overset{ˆ}{\psi }}_{i,w}(f)$, i=1,…,n, are the empirical versions of the influence functions. The latter are obtained by replacing expectations by sample averages and unknown parameters by their consistent estimates in $\psi_{i,w}(f)$. The formula for the empirical influence function is provided in Web Appendix B.

The final theorem characterizes the asymptotic distribution of the estimated value function of ${\overset{ˆ}{d}}_{n,w}=sgn({\overset{ˆ}{f}}_{n,w})$ and provides the basis for the calculation of simultaneous confidence intervals over the preference weight set $𝒲=\left\{w_{1},\ldots ,w_{M}\right\}$.

### Theorem 4.

Suppose that ℱ is the space of linear functions. Then, under assumptions A1–A3, conditions C1–C5 in Web Appendix A, the additional assumption that the optimal linear decision function ${\tilde{f}}_{w}(z)$ satisfies $P\left({\tilde{f}}_{w}(Z)=0\right)=0,\;w\in 𝒲$, and for $\lambda_{n}>0$ with $\lambda_{n}\to 0$ and $n\lambda_{n}\to \infty$, we have that

$$\underset{w\in 𝒲}{max}\;\left|G_{n,w}({\overset{ˆ}{f}}_{n,w})-G_{n,w}\left({\tilde{f}}_{w}\right)\right|=o_{p}(1).$$

The proof of Theorem 4 is given in Web Appendix A.4. The last theorem and Theorem 3 imply that $G_{n,w}({\overset{ˆ}{f}}_{n,w})$ is asymptotically normal with zero mean and variance $\sigma_{w}^{2}({\tilde{f}}_{w})$. This variance can be consistently (in probability) estimated by ${\overset{ˆ}{\sigma }}_{w}^{2}({\overset{ˆ}{f}}_{n,w})=n^{-1}\sum_{i=1}^{n}\;{\overset{ˆ}{\psi }}_{i,w}^{2}({\overset{ˆ}{f}}_{n,w})$. This result can be used for the calculation of (pointwise) confidence intervals and conducting hypothesis testing regarding the performance of the estimated ITR ${𝒱}_{w}(sgn({\overset{ˆ}{f}}_{n,w}))$ under the preference weight w. Theorems 3 and 4 can also be used for simultaneous inference. Define the vector $G_{n}\;:={\left(G_{n,w_{1}}({\overset{ˆ}{f}}_{n,w_{1}}),\ldots ,G_{n,w_{M}}({\overset{ˆ}{f}}_{n,w_{M}})\right)}^{\prime }$ and let $∥x{∥}_{\infty }:\;={max}_{1\leq l\leq M}\;\left|x_{l}\right|$ denote the maximum norm of a vector $x={\left(x_{1},\ldots ,x_{M}\right)}^{\prime }\in R^{M}$. Theorems 3 and 4, the Cramér–Wold theorem, and the continuous mapping theorem, imply that, for any fixed matrix Q (of appropriate dimension),

$${∥QG_{n}∥}_{\infty }\overset{d}{\to }∥QG{∥}_{\infty },$$

where G∼N(0,Ω), with Ω being a positive semidefinite matrix with elements $\omega_{l,l}=\sigma_{w_{l}}^{2}({\tilde{f}}_{w_{l}}),\;l=1,\ldots ,M$, and $\omega_{l,l^{\prime }}=E\{\psi_{1,w_{l}}({\tilde{f}}_{w_{l}})\psi_{1,w_{l^{\prime }}}({\tilde{f}}_{w_{l^{\prime }}})\},\;l\neq l^{\prime }$. The covariance matrix Ω can be consistently (in probability) estimated by the matrix ${\overset{ˆ}{\Omega }}_{n}$ with elements ${\overset{ˆ}{\omega }}_{l,l}={\overset{ˆ}{\sigma }}_{w_{l}}^{2}({\overset{ˆ}{f}}_{n,w}),\;l=1,\ldots ,M$, and ${\overset{ˆ}{\omega }}_{l,l^{\prime }}=n^{-1}\sum_{i=1}^{n}\;{\overset{ˆ}{\psi }}_{i,w_{l}}({\overset{ˆ}{f}}_{n,w_{l}})\psi_{i,w_{l^{\prime }}}({\overset{ˆ}{f}}_{n,w_{l^{\prime }}}),\;l\neq l^{\prime }$. Setting $Q=diag\left(\sigma_{w_{1}}^{-1}\left({\tilde{f}}_{w_{1}}\right),\ldots ,\sigma_{w_{M}}^{-1}\left({\tilde{f}}_{w_{M}}\right)\right)$ implies that

$$\underset{w\in 𝒲}{max}\;|\sigma_{w}^{-1}({\tilde{f}}_{w})G_{n,w}({\overset{ˆ}{f}}_{n,w})|\overset{d}{\to }∥QG{∥}_{\infty }.$$

The last result implies that 1-α simultaneous confidence intervals over 𝒲, which account for the multiplicity due to considering multiple preference weights, can be calculated as

$$\left[{\hat{𝒱}}_{n,w}(sgn({\overset{ˆ}{f}}_{n,w}))-c_{\alpha }\frac{{\overset{ˆ}{\sigma }}_{w}({\overset{ˆ}{f}}_{n,w})}{\sqrt{n}},{\hat{𝒱}}_{n,w}(sgn({\overset{ˆ}{f}}_{n,w}))+c_{\alpha }\frac{{\overset{ˆ}{\sigma }}_{w}({\overset{ˆ}{f}}_{n,w})}{\sqrt{n}}\right],$$

w∈𝒲,

where $c_{a}$ is the 1-α percentile of the distribution of $∥QG{∥}_{\infty }$. This percentile can be easily estimated using the following simulation procedure. First, choose a large number B (say B=1,000) and simulate vectors $G_{b}∼N(0,{\overset{ˆ}{\;\Omega }}_{n})$, for b=1,…,B. Then, $c_{\alpha }$ can be estimated as the empirical 1-α percentile of the sample ${∥{\overset{ˆ}{Q}}_{n}G_{1}∥}_{\infty },\ldots ,{∥{\overset{ˆ}{Q}}_{n}G_{B}∥}_{\infty }$, where ${\overset{ˆ}{Q}}_{n}=diag({\overset{ˆ}{\sigma }}_{w_{1}}^{-1}({\overset{ˆ}{f}}_{n,w_{1}}),\ldots ,{\overset{ˆ}{\sigma }}_{w_{M}}^{-1}({\overset{ˆ}{f}}_{n,w_{M}}))$. Simultaneous confidence intervals for the differences ${𝒱}_{w}(sgn({\overset{ˆ}{f}}_{n,w}))-{𝒱}_{w}(1)$ and ${𝒱}_{w}(sgn({\overset{ˆ}{f}}_{n,w}))-{𝒱}_{w}(-1)$, w∈𝒲, where ${𝒱}_{w}(1)$ and ${𝒱}_{w}(-1)$ are the value functions for the fixed rules d(z):=1 and d(z):=-1, can be calculated similarly by expanding the vector $G_{n}$ to include $G_{n,w}(1)$ and $G_{n,w}(-1)$, w∈𝒲, and using an appropriate matrix Q. This is illustrated in the data application presented in Section 5. We argue that the same inference procedures can also be used for the jackknife value estimator ${\hat{𝒱}}_{n,w}^{jk}(sgn({\overset{ˆ}{f}}_{n,w})),\;w\in 𝒲$. This statement is justified numerically in the simulation studies.

## 4 | SIMULATION STUDIES

The finite sample performance of the proposed methods was evaluated in a series of simulation experiments. Specifically, we assessed the performance of (i) the proposed ITR estimator ${\overset{ˆ}{d}}_{n,w}$ and (ii) the proposed inference methods for the (true) value of the estimated ITR ${𝒱}_{w}({\overset{ˆ}{d}}_{n,w})$. We considered a binary treatment variable A∈{-1,1}, a two-dimensional covariate vector $Z={\left(Z_{1},Z_{2}\right)}^{\prime }$, and a multistate process {X(t):t∈[0,τ]} under a progressive illness–death model with state space S={1,2,3}. State 1 represented the initial disease state, state 2 the tumor response state, and state 3 the disease progression or death state, in a hypothetical oncology trial. We considered the preference weights $w_{1}=(0,\;1,\;0{)}^{\prime }$ and $w_{2}=(1,\;1,\;0{)}^{\prime }$, which correspond to the (restricted) mean duration of tumor response and the PFS time, respectively. Treatment was simulated with P(A=1)=0.5, while the covariates were simulated from the uniform distribution U(-1,1). The multistate process was simulated, conditionally on A and Z, based on the transition intensities $\;\alpha_{12}(A,Z)=exp(-0.5Z_{1}+0.5Z_{2}+Af_{w}^{*}(Z)),\;\alpha_{13}(A,Z)=exp(-0.5Z_{1}+0.5Z_{2})/4$, and $\;\alpha_{23}(A,Z)=exp(-0.5Z_{1}+0.5Z_{2}-Af_{w}^{*}(Z))/2$, where $\alpha_{hj}(a,z)$ represents the transition rate from state h to state j for a patient with ${\left(A,Z^{\prime }\right)}^{\prime }={\left(a,z^{\prime }\right)}^{\prime }$ and $f_{w}^{*},\;w\in \left\{w_{1},w_{2}\right\}$, is the optimal decision function under w. Under the aforementioned choices, $sgn(f_{w_{1}}^{*})=sgn(f_{w_{2}}^{*})=sgn(f_{w}^{*})$ (more details on this equality are provided in Web Appendix C). In total, we considered four scenarios according to the form of the optimal decision function as follows:

- Scenario 1: $f_{w}^{*}(Z)=Z_{1}+Z_{2}$
- Scenario 2: $f_{w}^{*}(Z)=2\left(Z_{1}-Z_{2}\right)$
- Scenario 3: $f_{w}^{*}(Z)=1+Z_{2}-exp\left(-Z_{1}\right)$
- Scenario 4: $f_{w}^{*}\left(Z\right)=2\;log\left(2-Z_{1}-Z_{2}\right)-1.4$

The right censoring time was simulated independently of the multistate process from the exponential distribution Exp⁡(θ), with θ∈{-1.6,-1,-0.4}, and the total duration of the study was set to τ=3. These choices for θ and τ led on average to 28.4%, 42.8%, and 59.5% right-censored observations, respectively.

For each scenario and censoring rate θ, we considered the training sample sizes n∈{100,200,300,400} and repeated the simulation 1,000 times. In each training dataset, we applied the proposed method with the search space ℱ being the space of linear functions and τ=3. Note that, $f_{w}^{*}\notin ℱ$ in Scenarios 3 and 4. The tuning parameter was set to $\lambda_{n}=n^{-1/2}$, which satisfies the requirements of Theorems 2 and 4. To evaluate the performance of the estimated ITR ${\overset{ˆ}{d}}_{n,w}$, we considered two metrics: (i) the estimated ITR value ratio ${𝒱}_{w}({\overset{ˆ}{d}}_{n,w})/{𝒱}_{w}(d_{w}^{*})$, where $d_{w}^{*}=sgn(f_{w}^{*})$ and ${𝒱}_{w}(d_{w}^{*})$ is the maximum possible value, and (ii) the misclassification rate, defined as the proportion of patients which were assigned by ${\overset{ˆ}{d}}_{n,w}$ to the wrong treatment. An estimated ITR value ratio close to 1 indicates that the performance of ${\overset{ˆ}{d}}_{n,w}$ is close to optimal. For each simulated training dataset, the true value of the estimated ITR ${𝒱}_{w}({\overset{ˆ}{d}}_{n,w})$ and the misclassification rate were calculated based on an independently simulated large testing dataset of size 10,000. To evaluate the validity of the proposed inference methods for ${𝒱}_{w}({\overset{ˆ}{d}}_{n,w})$ we considered: (i) the average percent errors of the value function estimators ${\hat{𝒱}}_{n,w}({\overset{ˆ}{d}}_{n,w})$ and ${\hat{𝒱}}_{n,w}^{jk}({\overset{ˆ}{d}}_{n,w})$, defined as

$$\begin{array}{l} \frac{1}{1000}\sum_{b=1}^{1000}\;\frac{{\hat{𝒱}}_{n,w,b}({\overset{ˆ}{d}}_{n,w,b})\;-\;{𝒱}_{w}({\overset{ˆ}{d}}_{n,w,b})}{{𝒱}_{w}({\overset{ˆ}{d}}_{n,w,b})}\times 100\;\text{and}\;\\ \frac{1}{1000}\sum_{b=1}^{1000}\;\frac{{\hat{𝒱}}_{n,w,b}^{jk}({\overset{ˆ}{d}}_{n,w,b})\;-\;{𝒱}_{w}({\overset{ˆ}{d}}_{n,w,b})}{{𝒱}_{w}({\overset{ˆ}{d}}_{n,w,b})}\times 100, \end{array}$$

where ${\hat{𝒱}}_{n,w,b},\;{\hat{𝒱}}_{n,w,b}^{jk}$, and ${\overset{ˆ}{d}}_{n,w,b}$ are estimates from the bth simulated training dataset, (ii) the average of the proposed standard error estimates relatively to the Monte Carlo standard deviation of the value estimates, and (iii) the coverage probability of the 95% confidence intervals calculated using the proposed standard error estimator under asymptotic normality.

**FIGURE 1:**
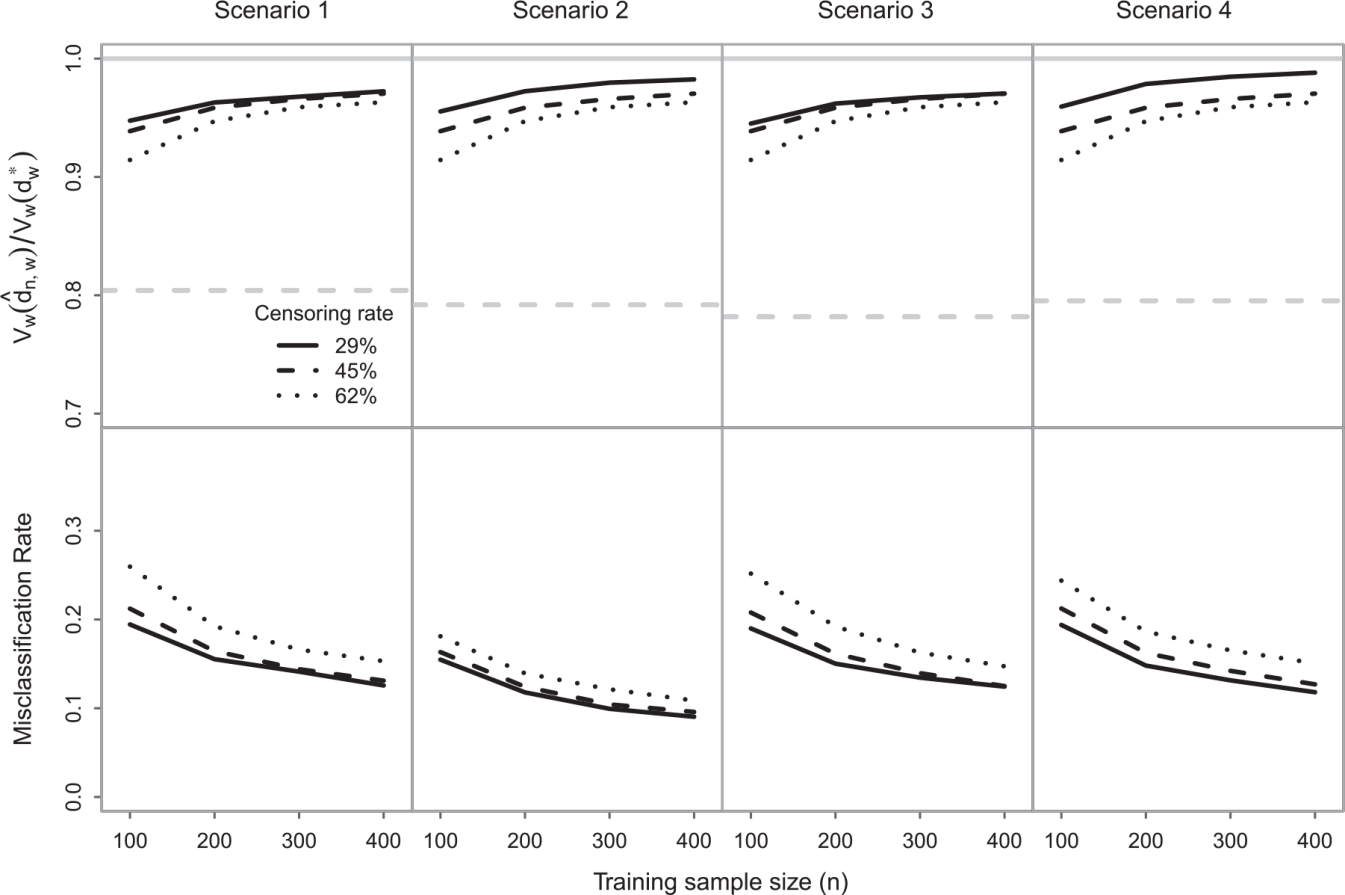
Simulation study: performance of ${\overset{ˆ}{d}}_{n,w}$ for the duration of tumor response (i.e., $w=(0,\;1,\;0{)}^{\prime }$, in terms of the average estimated ITR value ratio ${𝒱}_{w}({\overset{ˆ}{d}}_{n,w})/{𝒱}_{w}(d_{w}^{*})$ (top row) and misclassification rate (bottom row). The solid grey horizontal lines (top row) correspond to an optimal performance while the dashed grey horizontal lines (top row) correspond to the maximum fixed rule value ratio $max\left\{{𝒱}_{w}(1),{𝒱}_{w}(-1)\right\}/{𝒱}_{w}(d_{w}^{*})$, where ${𝒱}_{w}(1)$ and ${𝒱}_{w}(-1)$ are the values for the fixed rules $d\left(z\right):\;=1$ and $d\left(z\right):\;=-1$. ITR, individualized treatment rule.

The simulation results regarding the performance of the estimated ITR ${\overset{ˆ}{d}}_{n,w}$ for the time in response (i.e., $w=(0,\;1,\;0{)}^{\prime }$) are depicted in Figure 1. The estimated ITR value ratio was above 0.9 in all cases. Thus, even in Scenarios 3 and 4, where $f_{w}^{*}$ is not linear, the performance of the estimated rule was close to optimal. The maximum fixed rule value ratio $max\left\{{𝒱}_{w}(1),{𝒱}_{w}(-1)\right\}/{𝒱}_{w}(d_{w}^{*})$, where ${𝒱}_{w}(1)$ and ${𝒱}_{w}(-1)$ are the values for the fixed rules $d\left(z\right):=1$ and $d\left(z\right):\;=-1$, was close to 0.8 in all cases. This indicates that the estimated ITR ${\overset{ˆ}{d}}_{n,w}$ can lead to substantially better health outcomes on average compared to fixed, one-size-fits-all, rules. As expected, the estimated ITR value ratio was higher with larger training sample sizes and lower censoring rates. A similar pattern was observed for the misclassification rate of ${\overset{ˆ}{d}}_{n,w}$, which was lower for larger training samples and lower censoring rates. The simulation results regarding the validity of the proposed inference methods for ${𝒱}_{w}({\overset{ˆ}{d}}_{n,w})$ are summarized in Tables 1 and 2. In all cases, the value estimator ${\hat{𝒱}}_{n,w}({\overset{ˆ}{d}}_{n,w})$ provided slightly optimistic estimates of the true value ${𝒱}_{w}({\overset{ˆ}{d}}_{n,w})$. The percent error was over 4% only in a few cases with n=100 and a 60% censoring rate. As expected, the percent error of the jackknife value estimator ${\hat{𝒱}}_{n,w}^{jk}({\overset{ˆ}{d}}_{n,w})$ was lower than that of ${\hat{𝒱}}_{n,w}({\overset{ˆ}{d}}_{n,w})$. However, the difference between the two value estimators was smaller for larger training samples and lower censoring rates. The average standard error estimates were close to the corresponding Monte Carlo standard deviation of the estimates and the coverage probabilities close to the nominal level in all cases. These results indicate the consistency of the proposed standard error estimator and support the asymptotic normality result from Theorems 3 and 4.

Additional simulation results evaluating the effect of selecting different values of τ for the analysis are presented in Figures 1–3 in Web Appendix C.1. In these simulation studies, we considered the values τ∈{1,2,3}, with 3 being equal to the length of the study. In these studies, larger values of τ led to a better performance in terms of both the median value function and the variability. This is to be expected as more information is incorporated in the proposed method with a larger value of τ. However, the differences between the choices τ=2 and τ=3 were not pronounced in general. Further simulation results on the performance of the proposed ITR estimator when ℱ is the RKHS with the Gaussian kernel with σ=1 (less flexible kernel) and σ=5 (more flexible kernel), for the duration of tumor response, are presented in Figures 4– 6 in Web Appendix C.1. A larger training sample n led to a better performance in all cases, which reflects the consistency of the proposed ITR estimator. Using a more flexible kernel led to an inferior performance with smaller training sample sizes n. In most cases with n=200 or n=400, the performance of a less flexible kernel was the best, while the use of a linear decision function led in general to a better performance compared to two kernel choices when n=100. However, in many cases, the differences between a less flexible kernel and the linear decision function were not pronounced.

Simulation results regarding the performance of the estimated ITR ${\overset{ˆ}{d}}_{n,w}$ for the PFS time (i.e., $w=(1,\;1,\;0{)}^{\prime }$) are depicted in Figures 7– 9 in Web Appendix C.2. For comparison, these plots also illustrate the performance of the ICO and DR methods (Zhao et al., 2015). To apply the DR approach, we estimated E(T∣T>t,A,Z) based on the semiparametric Cox model for T with A,Z, and the interactions between A and Z as covariates, according to Zhao et al. (2015). This model is misspecified due to the complexity of the multistate process considered in the simulation studies. The performance of all methods improved with a larger sample size. Furthermore, the proposed method provided estimated ITRs with a substantially lower variability and slightly larger median value (i.e., PFS time) compared to the ICO and DR methods. The improved performance of the proposed method over the ICO and DR methods was more pronounced with a higher censoring rate. The simulation results regarding the validity of the proposed inference methods for ${𝒱}_{w}({\overset{ˆ}{d}}_{n,w})$ are summarized in Tables 1 and 2 in Web Appendix C.2. Results on the ICO and DR methods are not reported, there are no such inference procedures for these methods. Similarly to the simulations for the time in response, the performance of our inference methods was satisfactory in all cases, with the exception of low coverage probabilities for the jackknife estimator when n=100. The latter coverage probabilities were at the nominal level when n=400. Further simulation results for evaluating the performance of the proposed ITR estimator when ℱ is the RKHS with the Gaussian kernel are illustrated in Figures 10– 12 in Web Appendix C.2. These results revealed similar patterns to those for the duration of the tumor response.

## 5 | SPECTRUM TRIAL DATA ANALYSIS

The proposed methodology was applied to data from the SPECTRUM Trial (Vermorken et al., 2013), a phase III randomized trial on recurrent or metastatic squamous-cell carcinoma of the head and neck. The goal of this trial was to evaluate the effectiveness of the addition of panitumumab, a fully human monoclonal antibody which inhibits the epidermal growth factor receptor, to chemotherapy as a first-line treatment approach. Of the 520 patients included in this analysis, 260 patients were randomly assigned to the chemotherapy+panitumumab group (A=1), while the remaining patients were assigned to the chemotherapy alone group (A=-1). In this trial, tumor response was defined as an at least 30% decrease in the sum of the longest diameter of target lesions according to the response evaluation criteria in solid tumors (Therasse et al., 2000). Throughout the follow-up period, 138 (26.5%) patients achieved tumor response, while 457 (87.9%) experienced a progression of their disease and/or died. Among the latter patients, 120 (26.3%) had achieved tumor response prior to their disease progression or death. The overall median (95% CI) PFS time was 5.59 (5.29, 5.85) months. The estimates of the treatment-specific cumulative transition intensities and state occupation probabilities are depicted in Figures 13 and 14 in Web Appendix D.

In this analysis, the value τ was set to 18 months (90th percentile of the follow-up times; there were not many transitions to or from the tumor response state after this timepoint) and the preference weight set was $𝒲=\left\{w_{1},w_{2},w_{3}\right\}=\left\{(0,\;1,\;0{)}^{\prime },(0.5,\;1,\;0{)}^{\prime },(1,\;1,\;0{)}^{\prime }\right\}$. As mentioned in Section 2, in this multistate process setup, the utility under the preference weight $w_{1}$ corresponds to the restricted mean duration of tumor response, while the choices $w_{2}$ and $w_{3}$ provide a restricted quality-adjusted lifetime (where the time spent in the initial state is reduced by 50%) and the restricted PFS time, respectively. The covariates considered in this analysis were centered age (in years) at randomization $\left(Z_{\text{age.c}}\right)$, indicator that the primary tumor site is hypopharynx $\left(Z_{\text{hyp}}\right)$, indicator of history of the prior treatment for squamous-cell carcinoma of the head and neck $\left(Z_{\text{trt.hist}}\right)$, and indicator that ECOG performance status at baseline indicates symptoms but the patient is ambulatory (vs. fully active; $Z_{ECOG}$). The tuning parameters $\lambda_{n,w},\;w\in 𝒲$, were selected from the candidate set $\{0.001,\;0.005,\;0.01,\;0.1,\;0.25,\;0.5,\;1,\;2.5,\;5,\;10,\;20,\;50,\;100\}\times n^{-1/2}$, where n=520, using leave-one-out cross-validation. All these potential choices for $\lambda_{n,w}$ satisfy the requirements of the theorems in Section 3. The estimated optimal decision functions were

$$\begin{array}{l} {\overset{ˆ}{f}}_{n,w_{1}}(z)=\;-1.32-0.54z_{\text{age.c}}+0.90z_{\text{hyp}}\\ \;+1.17z_{\text{trt.hist}}+0.83z_{ECOG}, \end{array}$$

${\overset{ˆ}{f}}_{n,w_{2}}(z)\approx -1.40+2.00z_{\text{trt.hist}}$, and ${\overset{ˆ}{f}}_{n,w_{3}}(z)\approx -1.47+2.00z_{\text{trt.hist}}$, where the absolute values of the estimated coefficients of $z_{\text{age.c}},\;z_{hyp}$, and $z_{ECOG}$, in ${\overset{ˆ}{f}}_{n,w_{2}}(z)$ and ${\overset{ˆ}{f}}_{n,w_{3}}(z)$ were all less than 10^−6^. The more complicated form of ${\overset{ˆ}{f}}_{n,w_{1}}$ may reflect that there is higher heterogeneity in the treatment effect on tumor response compared to the treatment effect on quality-adjusted lifetime and PFS time. Intuitively, one would expect that this higher heterogeneity with respect to tumor response would be carried over to the other outcomes. However, this may not be the case because response has typically a short duration for this tumor and might not have a substantial impact on disease progression and/or death. The class of estimated ITRs is $\left\{{\overset{ˆ}{d}}_{n,w}=\mathrm{sgn}\left({\overset{ˆ}{f}}_{n,w}\right):w\in \left\{w_{1},w_{2},w_{3}\right\}\right\}$. Clearly, the estimated ITRs ${\overset{ˆ}{d}}_{n,w_{2}}$ and ${\overset{ˆ}{d}}_{n,w_{3}}$ are equivalent and assign chemotherapy+panitumumab (treatment 1) to the patients with a history of prior treatment, and chemotherapy alone (treatment −1) to those without prior treatment. In contrast, the rule ${\overset{ˆ}{d}}_{n,w_{1}}$ is more complicated and accounts for more covariates. Next, we estimated the performances (i.e., value functions) of the estimated optimal ITRs and compared them to those of the fixed, one-size-fits-all, rules $d\left(z\right):=1$ (everyone is assigned to chemotherapy+panitumumab) and $d\left(z\right):=-1$ (everyone is assigned to chemotherapy alone). To account for the multiplicity due to conducting inference about nine parameters in total, we calculated 95% simultaneous confidence intervals using the approach described in Section 3. The percentile $c_{0.05}$ was estimated based on B=1,000 simulation replications and the corresponding estimate was ${\overset{ˆ}{c}}_{0.05}=2.59$. The results from this analysis are summarized in Table 3. The estimated restricted mean (95% CI) potential time in the tumor response state under ${\overset{ˆ}{d}}_{n,w_{1}}$ was 2.57 (1.76, 3.38) months. This time was significantly longer than the corresponding time under the fixed rule $d\left(z\right):=-1$ [difference (95% CI): 0.86 (0.03, 1.70) months]. The estimated restricted mean (95% CI) potential quality-adjusted lifetime under ${\overset{ˆ}{d}}_{n,w_{2}}$ was 5.00 (4.17, 5.83) months. This time was significantly longer than that under the fixed rule $d\left(z\right):\;=-1$ [difference (95% CI): 0.95 (0.00, 1.90) months]. Finally, the estimated restricted mean (95% CI) potential PFS time under ${\overset{ˆ}{d}}_{n,w_{3}}$ was 7.43 (6.38, 8.49) months. There were no significant differences in the restricted mean PFS time between the optimal rule and $d\left(z\right):\;=-1$ [difference (95% CI): 1.04 (−0.25, 2.33) months] and between $d\left(z\right):\;=1$ and $d\left(z\right):\;=-1$ [difference (95% CI): 0.97 (−0.27, 2.22) months]. Also, no significant differences were observed between the optimal rule and $d\left(z\right):\;=1$. This might be due to a potentially small difference between the effects of chemotherapy alone and chemotherapy+panitumumab among patients for which the two rules assigned different treatment.

## 6 | DISCUSSION

This paper addressed the issue of optimal ITR estimation in randomized clinical trials with right-censored multistate processes. To achieve this, we devised a novel objective function that can handle general nonhomogeneous multistate processes and can easily incorporate patient preferences. A key feature of the proposed methodology is that it utilizes information from both uncensored and censored observations without positing and estimating a model for the conditional expectation of the outcome given A and Z, as opposed to the methods by Zhao et al. (2015) for censored failure times. Optimization of this objective function was based on the outcome-weighted learning framework (Zhao et al., 2012). The simulation studies provided numerical evidence for the validity of the proposed ITR estimation approach and inference procedures. Also, these studies showed a better performance of the proposed method compared to the methods by Zhao et al. (2015) for censored failure times.

There are two important practical considerations when applying the proposed approach. First, one needs to specify the preference weight set 𝒲. This can be achieved by subject matter experts (e.g., clinicians) or via a survey in a sample of patients. For a given 𝒲, the choice of the most appropriate rule within the class of estimated ITRs $\left\{{\overset{ˆ}{d}}_{n,w}\;:\;w\in 𝒲\right\}$ for a given patient with a preference weight $w_{0}$ is ${\overset{ˆ}{d}}_{n,w}$, with $w=arg{min}_{w\in 𝒲}\;∥w-w_{0}∥$ (i.e., the closest preference weight in the set 𝒲). Second, the class of decision functions ℱ needs to be chosen. Even though the proposed ITR estimator is universally consistent when ℱ is the RKHS with the Gaussian kernel, the rate of convergence under this choice may be extremely slow (Steinwart & Christmann, 2008). This means that an extremely large-sample size may be needed in practice in order to obtain an ITR ${\overset{ˆ}{d}}_{n,w}$, whose value ${𝒱}_{w}({\overset{ˆ}{d}}_{n,w})$ is reasonably close to the optimal value ${𝒱}_{w}({\overset{ˆ}{d}}_{w}^{*})$. For this reason, we mainly focused on the class of linear decision functions in most of this paper. Under this choice, the proposed estimator is universally consistent only if the true optimal decision function $f_{w}^{*}$ is linear. However, even if $f_{w}^{*}$ is not linear, the value of the estimated ITR ${𝒱}_{w}({\overset{ˆ}{d}}_{n,w})$ can be close to the optimal value. This was illustrated in the simulation studies presented in Section 4. In practice, one can further improve the performance of the estimated ITR when ℱ is the class of linear functions by considering two-way or three-way covariate interactions (Zhou et al., 2017).

A key assumption of the proposed approach is independent censoring. This assumption is realistic in many clinical trials, where accrual time is not associated with patient characteristics and censoring is mainly due to administrative reasons (Goldberg & Kosorok, 2017; Zhao et al., 2011). A plausible relaxation of this assumption is to allow censoring to depend on treatment A, since censoring rate will likely be higher among those receiving the treatment with the greater toxicity (Templeton et al., 2020). A further relaxation of the independent censoring assumption is to allow censoring to depend on both A and Z by imposing a semiparametric Cox model (more details on both relaxations are provided in Web Appendix E). It must be noted that, for the case of censored failure times, the DR method (Zhao et al., 2015) allows the censoring model to be misspecified (unlike the proposed approach) provided that the failure time model is correctly specified. However, the true censoring model may be of a less complicated form than the true failure time model in clinical trial applications (Goldberg & Kosorok, 2017) and, thus, more likely to be correctly specified.

An interesting extension of this work is to allow for interval censoring. This could be achieved by utilizing an estimator of the state occupation probabilities with interval-censored data, and deriving an appropriate objective function using, potentially, calculations similar to those in Section 2. Also, extending the proposed approach for the single-decision problem to the multi-decision setting, such as a sequential multiple assignment randomized trial (SMART) (Lavori & Dawson, 2000), is both practically and methodologically important.

## Supplementary Material

Supplement

R code description

R code

Example dataset

## Figures and Tables

**TABLE 1:** Simulation study: performance of the proposed inference methods for the true value of the estimated ITR ${𝒱}_{w}({\overset{ˆ}{d}}_{n,w})$ for the duration of tumor response (i.e., $\left.w=(0,1,0{)}^{\prime }\right)$, under a linear optimal decision function $f_{w}^{*}$ (Scenarios 1 and 2).

Scenario	Cens	*n*	${\overset{ˆ}{𝒱}}_{n,w}({\overset{ˆ}{d}}_{n,w})$				${\overset{ˆ}{𝒱}}_{n,w}^{jk}({\overset{ˆ}{d}}_{n,w})$			
			*%* error	MCSD	ASE	CP	*%* error	MCSD	ASE	CP

1	28%	100	3.418	0.224	0.215	0.954	−1.402	0.226	0.215	0.940
		200	2.129	0.151	0.155	0.961	−0.540	0.153	0.155	0.953
		400	1.062	0.109	0.110	0.954	−0.511	0.112	0.110	0.936
	43%	100	4.707	0.249	0.240	0.955	−0.856	0.248	0.240	0.946
		200	2.731	0.171	0.173	0.951	−0.039	0.172	0.173	0.949
		400	1.330	0.117	0.123	0.966	−0.630	0.120	0.123	0.954
	60%	100	5.289	0.313	0.303	0.951	−0.602	0.301	0.303	0.951
		200	3.411	0.215	0.218	0.964	0.016	0.218	0.218	0.947
		400	1.983	0.141	0.154	0.964	−0.681	0.143	0.154	0.962
2	28%	100	2.393	0.265	0.231	0.947	−0.663	0.261	0.231	0.940
		200	1.359	0.172	0.166	0.953	−0.529	0.174	0.166	0.941
		400	0.807	0.123	0.118	0.964	−0.218	0.124	0.118	0.953
	42%	100	2.403	0.288	0.258	0.951	−1.570	0.286	0.258	0.939
		200	1.568	0.194	0.185	0.953	−0.692	0.198	0.185	0.944
		400	1.207	0.139	0.132	0.946	−0.078	0.140	0.132	0.945
	57%	100	1.164	0.353	0.319	0.935	−3.364	0.343	0.319	0.931
		200	1.953	0.242	0.230	0.953	−0.785	0.236	0.230	0.944
		400	1.217	0.162	0.164	0.964	−0.492	0.165	0.164	0.955

*Note:* Cens, right censoring rate; *𝑛*, training sample size; MCSD, Monte Carlo standard deviation of the estimates; ASE, average of the standard error estimates; CP: empirical coverage probability of the 95% confidence interval.

**TABLE 2:** Simulation study: performance of the proposed inference methods for the true value of the estimated individualized treatment rule ${𝒱}_{w}\left(d_{n,w}\right)$ for the duration of tumor response (i.e., $\left.w=(0,1,0{)}^{\prime }\right)$, under a nonlinear optimal decision function $f_{w}^{*}$ (Scenarios 3 and 4).

Scenario	Cens	*n*	${\overset{ˆ}{𝒱}}_{n,w}({\overset{ˆ}{d}}_{n,w})$				${\overset{ˆ}{𝒱}}_{n,w}^{jk}({\overset{ˆ}{d}}_{n,w})$			
			*%* error	MCSD	ASE	CP	*%* error	MCSD	ASE	CP

3	29%	100	3.706	0.240	0.216	0.958	−0.415	0.236	0.216	0.942
		200	2.242	0.160	0.156	0.963	−0.049	0.156	0.156	0.956
		400	1.136	0.113	0.111	0.956	−0.331	0.115	0.111	0.943
	43%	100	4.483	0.258	0.243	0.959	−0.999	0.265	0.243	0.935
		200	2.355	0.182	0.174	0.948	−0.563	0.184	0.174	0.937
		400	1.505	0.120	0.124	0.951	−0.264	0.124	0.124	0.949
	60%	100	4.719	0.317	0.305	0.949	−1.154	0.306	0.305	0.938
		200	3.421	0.220	0.219	0.962	0.254	0.214	0.219	0.957
		400	1.397	0.146	0.155	0.964	−0.967	0.143	0.155	0.961
4	29%	100	3.957	0.229	0.216	0.947	−0.411	0.234	0.216	0.945
		200	2.232	0.158	0.155	0.950	−0.269	0.160	0.155	0.944
		400	1.128	0.113	0.111	0.948	−0.380	0.115	0.111	0.936
	43%	100	4.419	0.262	0.240	0.947	0.260	0.268	0.240	0.927
		200	2.278	0.180	0.173	0.961	−0.786	0.181	0.173	0.948
		400	1.197	0.128	0.123	0.943	−0.648	0.129	0.123	0.932
	59%	100	5.189	0.303	0.306	0.962	−1.175	0.313	0.306	0.927
		200	2.862	0.224	0.218	0.952	−1.089	0.226	0.218	0.929
		400	1.526	0.161	0.154	0.949	−0.809	0.161	0.154	0.931

*Note:* Cens, right censoring rate; *𝑛*, training sample size; MCSD, Monte Carlo standard deviation of the estimates; ASE, average of the standard error estimates; CP, empirical coverage probability of the 95% confidence interval.

**TABLE 3:** Analysis of the SPECTRUM trial: estimated mean potential utilities (i.e., value functions) under the estimated optimal individualized treatment rules ${\overset{ˆ}{d}}_{n,w}$ for the preference weights $w\in \left\{(0,\;1,\;0{)}^{\prime },(0.5,\;1,\;0{)}^{\prime },(1,\;1,\;0{)}^{\prime }\right\}$, and comparison with those under the fixed, one-size-fits-all, rules d(z):=1 (everyone is assigned to chemotherapy+panitumumab) and d(z):=-1 (everyone is assigned to chemotherapy alone).

*w*	Parameter	Estímate	95% CI

(0, 1, 0)’	${𝒱}_{w}\;{\overset{ˆ}{d}}_{n,w})$	2.570	(1.763, 3.376)
	${𝒱}_{w}\;d_{n,w})-{𝒱}_{w}(1)$	0.109	(−0.481, 0.700)
	${𝒱}_{w}\;{\overset{ˆ}{d}}_{n,w})-{𝒱}_{w}(-1)$	0.862	(0.025, 1.699)
(0.5, 1, 0)’	${𝒱}_{w}\;{\overset{ˆ}{d}}_{n,w})$	4.999	(4.168, 5.829)
	${𝒱}_{w}\;d_{n,w})-{𝒱}_{w}(1)$	0.087	(−0.539, 0.713)
	${𝒱}_{w}\;{\overset{ˆ}{d}}_{n,w})-{𝒱}_{w}(-1)$	0.949	(0.003, 1.896)
(1, 1, 0)’	${𝒱}_{w}\;{\overset{ˆ}{d}}_{n,w})$	7.434	(6.377, 8.490)
	${𝒱}_{w}\;d_{n,w})-{𝒱}_{w}(1)$	0.071	(−0.739, 0.881)
	${𝒱}_{w}\;{\overset{ˆ}{d}}_{n,w})-{𝒱}_{w}(-1)$	1.043	(−0.248, 2.334)

Note: The corresponding 95% simultaneous confidence intervals, that adjust for multiplicity due to conducting inference about nine parameters in total, are also provided. $({𝒱}_{w}({\overset{ˆ}{d}}_{n,w})$, mean potential utility under ${\overset{ˆ}{d}}_{n,w}$ for the preference weight $w;{𝒱}_{w}(1)$, mean potential utility for the preference weight w under the fixed rule $d(z):=1;{𝒱}_{w}(-1)$, mean potential utility for the preference weight w under the fixed rule d(z):=-1).

## Data Availability

The data that support the findings of this paper are available from Project Data Sphere. Restrictions apply to the availability of these data, which were used under license in this paper. Data are available at https://www.projectdatasphere.org/ with the permission of Project Data Sphere.
