# Supplementary material for: Estimating optimal individualized treatment rules with multistate processes
Source: Biometrics. Author manuscript; Available in PMC 2024 Feb 16. (PMC10553793; doi:10.1111/biom.13864)
Supplement: Supplement [file NIHMS1931646-supplement-Supplement.pdf]

BIOMETRICS 000, 000–000 DOI: 000
000 0000

Supporting Information for "Estimating optimal individualized treatment rules with multistate processes" by

Giorgos Bakoyannis

Department of Biostatistics and Health Data Science, Indiana University, Indianapolis, Indiana, U.S.A.

email: gbakoyannis@gmail.com

This paper has been submitted for consideration for publication in *Biometrics*

Web Appendix A. Proofs of Theoretical Results

In this Web Appendix we provide the proofs of the theorems stated in Section 3 of the main text. The proposed methodology assumes the following regularity conditions.

C1. The right censoring time C is independent in the sense that $\{Y_w^*(\cdot;1), Y_w^*(\cdot;-1), A, Z\} \perp\!\!\!\perp C$.

C2. The benefit process has a square-integrable total variation, i.e. $E\{\int_0^\tau |dY_w(t)|\}^2 < \infty$.

C3. The covariate space $\mathcal{Z}$ is a compact subset of $\mathbb{R}^p$.

C4. The true cumulative baseline hazard $\Lambda_0(t)$ of the right censoring distribution is a continuous function on $[0, \tau]$.

C5. For a linear decision function $\tilde{f}_w(\cdot) = \tilde{\beta}_{0,w} + \langle \tilde{\beta}_{1,w}, \cdot \rangle$ that minimizes $\mathcal{R}_{\phi,w}(f)$ over the space of linear functions, $(\tilde{\beta}_{0,w}, \tilde{\beta}'_{1,w})' \in \mathcal{B} \subset \mathbb{R}^{p+1}$, where $\mathcal{B}$ is a compact and convex set. Moreover, letting $T_w^* = \int_0^\tau Y_w(t) I(C \geqslant T \wedge t) \mathrm{d}m(t)$,

$$P[T_w^* > 0, A = 1, \{1 - (\tilde{\beta}_{0,w} + \langle \tilde{\beta}_{1,w}, Z \rangle)\}\{1 - (\beta_0 + \langle \beta_1, Z \rangle)\} < 0]$$

$$+P[T_w^* > 0, A = -1, \{1 + (\tilde{\beta}_{0,w} + \langle \tilde{\beta}_{1,w}, Z \rangle)\}\{1 + (\beta_0 + \langle \beta_1, Z \rangle)\} < 0] > 0,$$

for any $(\beta_0, \beta_1')' \neq (\tilde{\beta}_{0,w}, \tilde{\beta}'_{1,w})'$.

Conditions C1, C2, and C4 are standard in the literature of nonparametric methods for survival and multistate process data. A plausible relaxation of condition C1 is to allow censoring to depend on treatment A, since censoring will likely be higher among those receiving the treatment with the greater toxicity (Templeton et al., 2020). This can be trivially incorporated into the proposed methodology by simply estimating nonparametrically the cumulative hazard of censoring separately for the two treatment groups. A further relaxation of the independent censoring assumption is to allow censoring to depend on both A and Z. In this case, one can impose a semiparametric Cox model of the form $\Lambda(t; A, Z) = \Lambda_0(t)\exp\{\theta'(A, Z')'\}$ for the right censoring time, and use the estimated conditional hazard

in the proposed objective function and value function estimators. Provided that this model is correctly specified, the theoretical properties of the proposed method still hold, with the exception that $\gamma_i(t)$ in $\psi_{i,w}(f)$ (see Web Appendix B) is replaced by the influence function of $\sqrt{n}\{\hat{\Lambda}_n(t)\exp(\hat{\theta}_n'(a, z')') - \Lambda(t; a, z)\}$ under partial likelihood estimation. Condition C3 is another common condition that ensures that the covariates are bounded. Condition C5 guarantees the uniqueness of the optimal linear decision function $\tilde{f}_w$. A similar condition has been previously used in the literature of (unweighted) support vector machines (Jiang et al., 2008).

For notational simplicity, we omit the subscript w, that corresponds to the preference weight, and use the more compact notations $Y(t)$, $Y^*(t;1)$, $Y^*(t;-1)$, $\mathcal{V}(d)$, $\hat{\mathcal{V}}_n(d)$, $\hat{f}_n$, $\hat{d}_n$, and $\psi(f)$, for the remainder of Web Appendix A. The proofs of Theorems 2–4 rely heavily on empirical process theory (van der Vaart and Wellner, 1996; Kosorok, 2008). For these proofs, we use the standard empirical process theory notation

$$\mathbb{P}_n f = \frac{1}{n}\sum_{i=1}^{n} f(D_i),$$

for any measurable function of the data $f : \mathcal{D} \mapsto \mathbb{R}$, where $\mathcal{D}$ is the sample space, and

$$Pf = \int_{\mathcal{D}} f \mathrm{d}P = E\{f(D)\},$$

where P is the true probability measure on the Borel σ-algebra on $\mathcal{D}$. Furthermore, for any function h in the space $D[0,\tau]$ of cadlag functions on $[0,\tau]$, we define the supremum norm $\|h\|_{[0,\tau]} = \sup_{t\in[0,\tau]} |h(t)|$. Also, we define the class of fixed functions

$$\mathcal{L}_\delta = \{\Lambda : \Lambda \in D[0,\tau] \text{ and is non-decreasing with } \Lambda(0) = 0, \|\Lambda - \Lambda_0\|_{[0,\tau]} < \delta\},$$

for some $\delta > 0$, and the data-dependent function

$$\xi_\Lambda(D) = \int_0^\tau \frac{Y(t)I(C \geqslant T \wedge t)}{\exp\{-\Lambda(\tilde{T} \wedge t)\}} \mathrm{d}m(t).$$

Before providing the proofs of the theorems listed in the main text, we state and prove a useful lemma.

Lemma 1: *If conditions C2 and C4 are satisfied, then the class of functions* $\{\xi_\Lambda : \Lambda \in \mathcal{L}_\delta\}$ *is* P*-Donsker.*

Proof. The class of functions $\{Y(t) : t \in [0, \tau]\}$ is P-Donsker as a consequence of condition C2 and Lemma 22.4 in Kosorok (2008). Also, recognizing that

$$I(C \geqslant T \wedge t) = 1 - I(C < T)I(C < t),$$

the class $\{I(C \geqslant T \wedge t) : t \in [0, \tau]\}$ is P-Donsker by Lemma 4.1 in (Kosorok, 2008). Next, see that the class $\mathcal{L}_\delta$ is uniformly bounded by $\Lambda_0(\tau) + \delta$, where $\Lambda_0(\tau) < \infty$ by condition C4, and consider the data-dependent function $f_\Lambda : \mathcal{D} \mapsto [0, \Lambda_0(\tau) + \delta]$ with $f_\Lambda(D) = \Lambda(\tilde{T})$, where $\tilde{T} \in [0, \tau]$ and $\Lambda \in \mathcal{L}_\delta$. Now, the class $\{f_\Lambda : \Lambda \in \mathcal{L}_\delta\}$ is P-Donsker as a consequence of Lemma 9.11 in Kosorok (2008), as is (trivially) the fixed class $\{\Lambda(t) : \Lambda \in \mathcal{L}_\delta, t \in [0, \tau]\}$. Thus, by Corollary 9.32 in Kosorok (2008) and the Lipschitz continuity of the exponential function on $[0, \Lambda_0(\tau) + \delta]$, the class $\{\exp\{f_\Lambda \wedge \Lambda(t)\} : \Lambda \in \mathcal{L}_\delta, t \in [0, \tau]\}$ is also P-Donsker. Given that the latter class is uniformly bounded by $\exp\{\Lambda_0(\tau) + \delta\}$ and that $\Lambda(\tilde{T} \wedge t) = \Lambda(\tilde{T}) \wedge \Lambda(t)$, it follows that the class $\{\zeta_{\Lambda,t} : \Lambda \in \mathcal{L}_\delta, t \in [0, \tau]\}$ with

$$\zeta_{\Lambda,t}(D) = \frac{Y(t)I(C > T \wedge t)}{\exp\{-\Lambda(\tilde{T} \wedge t)\}},$$

is P-Donsker by virtue of being a product of uniformly bounded P-Donsker classes. Now, the conclusion of Lemma 1 follows from the fact that

$$\left| \int_0^\tau \zeta_{\Lambda_1,t} \mathrm{d}m(t) - \int_0^\tau \zeta_{\Lambda_2,t} \mathrm{d}m(t) \right| \leqslant \sup_{t \in [0,\tau]} |\zeta_{\Lambda_1,t} - \zeta_{\Lambda_2,t}| \, \tau,$$

which implies continuity, and Lemma 15.10 in Kosorok (2008). □

A.1 *Proof of Theorem 1 (Fisher consistency)*

Using the same arguments to those used in Tsiatis et al. (2019) for the case of a general ITR, it can be easily shown that the optimal ITR in our case satisfies

$$\begin{aligned} d^*(z) &= \underset{a\in\{-1,1\}}{\arg\max}\, E\left\{ \int_0^\tau Y^*(t;a)\mathrm{d}m(t) \middle| Z=z \right\} \\ &= \mathrm{sgn}\left[E\left\{ \int_0^\tau Y^*(t;1)\mathrm{d}m(t) \middle| Z=z \right\} - E\left\{ \int_0^\tau Y^*(t;-1)\mathrm{d}m(t) \middle| Z=z \right\} \right], \quad (1) \end{aligned}$$

for all $z \in \mathcal{Z}$. Now, by Tonelli's theorem (Athreya and Lahiri, 2006) and given that

$$\frac{Y(t)I(C \geqslant T\wedge t)}{\exp\{-\Lambda_0(T\wedge t)\}} = \frac{Y(t)I(C \geqslant T\wedge t)}{\exp\{-\Lambda_0(\tilde{T}\wedge t)\}}, \qquad t\in[0,\tau],$$

as argued in the main text, the surrogate risk $\mathcal{R}_\phi$ can be expressed as

$$\begin{aligned} \mathcal{R}_\phi(f) &= \int_0^\tau E\left[\frac{Y(t)I(C\geqslant T\wedge t)\phi(Af(Z))}{\exp\{-\Lambda_0(T\wedge t)\}\{A\pi_0+(1-A)/2\}} \right] \mathrm{d}m(t) \\ &= \int_0^\tau E\left[\frac{Y(t)\phi(Af(Z))E\{I(C\geqslant T\wedge t)|T,Y(t),A,Z\}}{\exp\{-\Lambda_0(T\wedge t)\}\{A\pi_0+(1-A)/2\}} \right] \mathrm{d}m(t). \end{aligned}$$

Given that condition C1 implies that

$$E\{I(C\geqslant T\wedge t)|T,Y(t),A,Z\} = E\{I(C\geqslant T\wedge t)|T\} = \exp\{-\Lambda_0(T\wedge t)\}, \qquad t\in[0,\tau],$$

it follows that

$$\begin{aligned} \mathcal{R}_\phi(f) &= \int_0^\tau E\left[\frac{Y(t)\phi(Af(Z))}{A\pi_0+(1-A)/2} \right] \mathrm{d}m(t) \\ &= \int_0^\tau E\left\{ Y(t)I(A=1)\frac{\phi(f(Z))}{\pi_0} + Y(t)I(A=-1)\frac{\phi(-f(Z))}{1-\pi_0} \right\} \mathrm{d}m(t). \end{aligned}$$

Next, by a second application of Tonelli's theorem and assumptions A1–A3 we have

$$\begin{aligned}
\mathcal{R}_\phi(f) &= \int_0^\tau E\left\{Y^*(t;1)I(A=1)\frac{\phi(f(Z))}{\pi_0}\right\}\mathrm{d}m(t) \\
&\quad + \int_0^\tau E\left\{Y^*(t;-1)I(A=-1)\frac{\phi(-f(Z))}{1-\pi_0}\right\}\mathrm{d}m(t) \\
&= E\left[\left\{\int_0^\tau Y^*(t;1)\mathrm{d}m(t)\right\}I(A=1)\frac{\phi(f(Z))}{\pi_0}\right] \\
&\quad + E\left[\left\{\int_0^\tau Y^*(t;-1)\mathrm{d}m(t)\right\}I(A=-1)\frac{\phi(-f(Z))}{1-\pi_0}\right] \\
&= E\left[E\left\{\int_0^\tau Y^*(t;1)\mathrm{d}m(t)\middle| Z\right\}\phi(f(Z))\right. \\
&\quad \left. + E\left\{\int_0^\tau Y^*(t;-1)\mathrm{d}m(t)\middle| Z\right\}\phi(-f(Z))\right].
\end{aligned}$$

The function $\tilde{f}$ that minimizes

$$E\left\{\int_0^\tau Y^*(t;1)\mathrm{d}m(t)\middle| Z\right\}\phi(f(Z)) + E\left\{\int_0^\tau Y^*(t;-1)\mathrm{d}m(t)\middle| Z\right\}\phi(-f(Z))$$

$$\begin{aligned}
&= E\left\{\int_0^\tau Y^*(t;1)\mathrm{d}m(t)\middle| Z\right\}\max(0,1-f(Z)) \\
&\quad + E\left\{\int_0^\tau Y^*(t;-1)\mathrm{d}m(t)\middle| Z\right\}\max(0,1+f(Z)) \\
&= E\left\{\int_0^\tau Y^*(t;1)\mathrm{d}m(t)\middle| Z\right\}\{1-f(Z)\}I(f(Z)\leqslant -1) \\
&\quad + E\left\{\int_0^\tau Y^*(t;-1)\mathrm{d}m(t)\middle| Z\right\}\{1+f(Z)\}I(f(Z)\geqslant 1) \\
&\quad + \Bigg(E\left\{\int_0^\tau Y^*(t;1)\mathrm{d}m(t)\middle| Z\right\} + E\left\{\int_0^\tau Y^*(t;-1)\mathrm{d}m(t)\middle| Z\right\} \\
&\quad + \left[E\left\{\int_0^\tau Y^*(t;-1)\mathrm{d}m(t)\middle| Z\right\} - E\left\{\int_0^\tau Y^*(t;1)\mathrm{d}m(t)\middle| Z\right\}\right]f(Z)\Bigg) \\
&\quad \times I(-1<f(Z)<1),
\end{aligned}$$

also minimizes $\mathcal{R}_\phi$. The latter function is a (continuous) piecewise linear function which decreases strictly on $(-\infty,-1]$ and increases strictly on $[1,\infty)$, almost surely. Therefore, the minimizer should lie in $[-1,1]$, almost surely. Consequently, for any $z\in\mathcal{Z}$, if

$$E\left\{\int_0^\tau Y^*(t;1)dm(t)\middle| Z=z\right\} > E\left\{\int_0^\tau Y^*(t;-1)dm(t)\middle| Z=z\right\},$$

then $\tilde{f}(z)$ should be positive, and if

$$E\left\{\int_0^\tau Y^*(t;1)dm(t)\middle| Z=z\right\} < E\left\{\int_0^\tau Y^*(t;-1)dm(t)\middle| Z=z\right\},$$

then $\tilde{f}(z)$ should be negative. Consequently, by (1), $d^*(z) = \text{sgn}\{\tilde{f}(z)\}$ for all $z \in \mathcal{Z}$.

A.2 *Proof of Theorem 2*

First, define $g_\pi(D) = A\pi + (1-A)/2$ and

$$L_{f,\Lambda,\pi}(D) = \frac{\xi_\Lambda(D)}{g_\pi(D)}\phi(Af(Z)),$$

for any $f \in \mathcal{F}$ (where $\xi_\Lambda(D)$ was defined in the beginning of Web Appendix A), and note that $\mathcal{R}_\phi(f) = PL_{f,\Lambda_0,\pi_0}$ and $\hat{\mathcal{R}}_\phi(f) = \mathbb{P}_n L_{f,\hat{\Lambda}_n,\hat{\pi}_n}$. Now, we have that

$$\begin{aligned}\frac{Y(t)I(C \geqslant T\wedge t)}{\exp\{-\hat{\Lambda}_n(\tilde{T}\wedge t)\}} &\leqslant \exp\{\hat{\Lambda}_n(\tilde{T}\wedge t)\}\\ &\leqslant \exp\{\hat{\Lambda}_n(\tau)\},\end{aligned}$$

for any $t \in [0,\tau]$ and all $n \geqslant 1$. This fact, along with the uniform (outer) almost sure consistency of the Nelson–Aalen estimator (guaranteed by conditions C1 and C4), the facts that

$$\int_0^\tau \exp\{\hat{\Lambda}_n(\tau)\}\mathrm{d}m(t) = \tau\exp\{\hat{\Lambda}_n(\tau)\} < \infty,$$

for all $n \geqslant 1$ and

$$\int_0^\tau \exp\{\Lambda_0(\tau)\}\mathrm{d}m(t) = \tau\exp\{\Lambda_0(\tau)\} < \infty,$$

and the extended dominated converenge theorem (Athreya and Lahiri, 2006), lead to the conclusion that $\xi_{\hat{\Lambda}_n}(D) \overset{as}{\to} \xi_{\Lambda_0}(D)$. This result, the almost sure consistency of $\hat{\pi}_n$, and the continuous mapping theorem imply that

$$\max_{1\leqslant i\leqslant n}\left|\frac{\xi_{\hat{\Lambda}_n}(D_i)}{g_{\hat{\pi}_n}(D_i)} - \frac{\xi_{\Lambda_0}(D_i)}{g_{\pi_0}(D_i)}\right| = o_{as}(1). \tag{2}$$

Consequently,

$$\sup_{f\in\mathcal{F}}\left\{\max_{1\leqslant i\leqslant n}\left|L_{f,\hat{\Lambda}_n,\hat{\pi}_n}(D_i) - L_{f,\Lambda_0,\pi_0}(D_i)\right|\right\} = o_{as}(1), \tag{3}$$

Next, define

$$\tilde{f} \in \arg\min_{f \in \mathcal{F}} \mathcal{R}_\phi(f).$$

By the positivity of λ_n and the definition of $\hat{f}_n$, we have that

$$\begin{aligned}
\mathbb{P}_n L_{\hat{f}_n,\hat{\Lambda}_n,\hat{\pi}_n} &\leqslant \mathbb{P}_n L_{\hat{f}_n,\hat{\Lambda}_n,\hat{\pi}_n} + \lambda_n \|\hat{f}_n\|^2 \\
&\leqslant \mathbb{P}_n L_{\tilde{f},\hat{\Lambda}_n,\hat{\pi}_n} + \lambda_n \|\tilde{f}\|^2 \\
&= \mathbb{P}_n L_{\tilde{f},\Lambda_0,\pi_0} + \lambda_n \|\tilde{f}\|^2 + o_{as}(1)
\end{aligned}$$

Taking limit superiors in both sides we get

$$\limsup_{n\to\infty} \mathbb{P}_n L_{\hat{f}_n,\hat{\Lambda}_n,\hat{\pi}_n} \leqslant PL_{\tilde{f},\Lambda_0,\pi_0} = \mathcal{R}_\phi(\tilde{f}),$$

almost surely, by the strong law of large numbers and the fact that $\lambda_n \to 0$. This implies that, fall all n sufficiently large,

$$\mathbb{P}_n L_{\hat{f}_n,\hat{\Lambda}_n,\hat{\pi}_n} \leqslant \mathcal{R}_\phi(\tilde{f}) \leqslant PL_{\hat{f}_n,\Lambda_0,\pi_0} = \mathcal{R}_\phi(\hat{f}_n),$$

almost surely, by the definition of $\tilde{f}$. Therefore, for all n sufficiently large, we have

$$\begin{aligned}
|\mathcal{R}_\phi(\hat{f}_n) - \mathcal{R}_\phi(\tilde{f})| &\leqslant \left|\mathbb{P}_n L_{\hat{f}_n,\hat{\Lambda}_n,\hat{\pi}_n} - PL_{\hat{f}_n,\Lambda_0,\pi_0}\right| \\
&\leqslant \left|(\mathbb{P}_n - P)L_{\hat{f}_n,\Lambda_0,\pi_0}\right| + \mathbb{P}_n \left|L_{\hat{f}_n,\hat{\Lambda}_n,\hat{\pi}_n} - L_{\hat{f}_n,\Lambda_0,\pi_0}\right| \\
&\leqslant \left|(\mathbb{P}_n - P)L_{\hat{f}_n,\Lambda_0,\pi_0}\right| + \delta, \qquad (4)
\end{aligned}$$

almost surely, for any $\delta > 0$ by (3). Next, it needs to be shown that $(\mathbb{P}_n - P)L_{\hat{f}_n,\Lambda_0,\pi_0} = o_p(1)$. By the definition of $\hat{f}_n$ we have,

$$\mathbb{P}_n L_{\hat{f}_n,\hat{\Lambda}_n,\hat{\pi}_n} + \lambda_n \|\hat{f}_n\|^2 \leqslant \mathbb{P}_n L_{f,\hat{\Lambda}_n,\hat{\pi}_n} + \lambda_n \|f\|^2,$$

for any $f \in \mathcal{F}$. Selecting $f \equiv 0$, we have

$$\mathbb{P}_n L_{\hat{f}_n,\hat{\Lambda}_n,\hat{\pi}_n} + \lambda_n \|\hat{f}_n\|^2 \leqslant \mathbb{P}_n \frac{\xi_{\hat{\Lambda}_n}}{g_{\hat{\pi}_n}},$$

since $\phi(0) = 1$ and $\|0\| = 0$. By the non-negativity of $L_{\hat{f}_n,\hat{\Lambda}_n,\hat{\pi}_n}(D)$, the previous inequality implies that

$$\begin{aligned}\lambda_n\|\hat{f}_n\|^2 &\leqslant \mathbb{P}_n\frac{\xi_{\hat{\Lambda}_n}}{g_{\hat{\pi}_n}} \\ &= \mathbb{P}_n\frac{\xi_{\Lambda_0}}{g_{\pi_0}} + o_{as}(1) \\ &\leqslant \frac{1}{n}\sum_{i=1}^{n}\frac{\tau\exp\{\Lambda_0(\tau)\}}{A_i\pi_0 + (1-A_i)/2} + o_{as}(1) \\ &\leqslant \frac{\tau\exp\{\Lambda_0(\tau)\}}{c_1 \wedge (1-c_2)} + o_{as}(1),\end{aligned}$$

by assumption A3. Therefore, for all sufficiently large n and any $\delta' > 0$, we have

$$\lambda_n\|\hat{f}_n\|^2 \leqslant \frac{\tau\exp\{\Lambda_0(\tau)\}}{c_1 \wedge (1-c_2)} + \delta' \equiv M_{\delta'},$$

almost surely. Now, the class of functions

$$\mathcal{G}_1(\delta') = \{\sqrt{\lambda_n}f : f \in \mathcal{F}, \|\sqrt{\lambda_n}f\| \leqslant \sqrt{M_{\delta'}}\}$$

is Donsker, for any $\delta' > 0$ small. This follows from the fact that if $\mathcal{F}$ is the class of linear or polynomial functions, then $\mathcal{F}$ is Donsker by Lemma 9.6 and Theorem 9.2 in Kosorok (2008), and subsets of Donsker class are also Donsker. If $\mathcal{F}$ is a RKHS, then the $\mathcal{G}_1(\delta')$ is Donsker by condition C3 and similar arguments to those used in the proof of Lemma A.9 in Hable (2012). Also, the class

$$\mathcal{G}_2(\delta') = \{\sqrt{\lambda_n}L_{f,\Lambda_0,\pi_0} : f \in \mathcal{F}, \|\sqrt{\lambda_n}f\| \leqslant \sqrt{M_{\delta'}}\}$$

is Donsker by Corollary 9.32 in Kosorok (2008), since L_{f,Λ_0,π_0} is Lipschitz continuous in f, since the hinge loss is Lipschitz continuous in f. This implies that, for all n sufficiently large, $\sqrt{\lambda_n}L_{\hat{f}_n;\Lambda_0,\pi_0}$ belongs to the Donsker class $\mathcal{G}_2(\delta')$ and therefore

$$\sqrt{n}(\mathbb{P}_n - P)\sqrt{\lambda_n}L_{\hat{f}_n,\Lambda_0,\pi_0} = O_p(1).$$

Consequently,

$$(\mathbb{P}_n - P)L_\phi(\hat{f}_n;\Lambda_0,\pi_0) = \frac{1}{\sqrt{n\lambda_n}}O_p(1) = o_p(1),$$

since $n\lambda_n \to \infty$ by assumption. Substituting this result in (4) we have

$$|\mathcal{R}_\phi(\hat{f}_n) - \mathcal{R}_\phi(\tilde{f})| \leqslant o_p(1) + \delta.$$

Setting $\delta = \delta_n \downarrow 0$, with $\sqrt{n}\delta_n \to \infty$, implies that

$$|\mathcal{R}_\phi(\hat{f}_n) - \mathcal{R}_\phi(\tilde{f})| = o_p(1),$$

which completes the proof of the first statement of Theorem 2. For the second statement, it can be shown using similar arguments to those used in the proof of Theorem 3.2 in Zhao et al. (2012) that

$$\mathcal{R}(f) - \mathcal{R}(f^*) \leqslant \mathcal{R}_\phi(f) - \mathcal{R}_\phi(f^*),$$

for any distribution P of the data D and any measurable decision function $f : \mathcal{Z} \mapsto \mathbb{R}$. Therefore,

$$\begin{aligned} |\mathcal{V}(\hat{d}_n) - \mathcal{V}(d^*)| &= |\mathcal{R}(\hat{f}_n) - \mathcal{R}(f^*)| \\ &\leqslant |\mathcal{R}_\phi(\hat{f}_n) - \mathcal{R}_\phi(f^*)|. \end{aligned} \tag{5}$$

Now, if $\mathcal{F}$ is the space of linear functions and $f^* \in \mathcal{F}$, then $\inf_{f\in\mathcal{F}} \mathcal{R}_\phi(f) = \mathcal{R}_\phi(\tilde{f}) = \mathcal{R}_\phi(f^*)$. Thus, consistency follows from the first statement of Theorem 2 and (5). Next, suppose that $\mathcal{F}$ is the RKHS with the Gaussian kernel and that the marginal distribution μ of Z is regular. Since the Gaussian kernel is a universal kernel, using similar arguments to those used in the proof of Lemma 3.4 in Zhou et al. (2017) leads to

$$\inf_{f\in\mathcal{F}} \mathcal{R}_\phi(f) = \mathcal{R}_\phi(f^*).$$

This, (5), and the first statement of Theorem 2 imply universal consistency.

A.3 *Proof of Theorem 3*

Here we provide the proof of the stronger (uniform) statement of Theorem 3 for the case where $\mathcal{F}$ is the space of linear functions. The proof of the weaker (pointwise) statement

for any given measurable function f is a simplified version of the proof below and is not discussed further.

First, note that, for any $f \in \mathcal{F}$, we have

$$\mathcal{V}(\text{sgn}(f)) = P\frac{\xi_{\Lambda_0}}{g_{\pi_0}}u_f,$$

where

$$u_f(D) = I\{A = \text{sgn}(f(Z))\} = I\{Af(Z) \geqslant 0\},$$

and

$$\hat{\mathcal{V}}_n(\text{sgn}(f)) = \mathbb{P}_n\frac{\xi_{\hat{\Lambda}_n}}{g_{\hat{\pi}_n}}u_f.$$

Now, straightforward algebra leads to

$$\begin{aligned}
\sqrt{n}\left\{\hat{\mathcal{V}}_n(\text{sgn}(f)) - \mathcal{V}(\text{sgn}(f))\right\} &= \sqrt{n}\mathbb{P}_n\left(\frac{1}{g_{\hat{\pi}_n}} - \frac{1}{g_{\pi_0}}\right)\left(\xi_{\hat{\Lambda}_n} - \xi_{\Lambda_0}\right)u_f \\
&\quad + \sqrt{n}\mathbb{P}_n\frac{\xi_{\hat{\Lambda}_n} - \xi_{\Lambda_0}}{g_{\pi_0}}u_f \\
&\quad + \sqrt{n}\mathbb{P}_n\xi_{\Lambda_0}\left(\frac{1}{g_{\hat{\pi}_n}} - \frac{1}{g_{\pi_0}}\right)u_f \\
&\quad + \sqrt{n}(\mathbb{P}_n - P)\frac{\xi_{\Lambda_0}}{g_{\pi_0}}u_f \\
&\equiv B_{n,1}(f) + B_{n,2}(f) + B_{n,3}(f) + B_{n,4}(f) \qquad (6)
\end{aligned}$$

Next, for any functional $h : \mathcal{F} \mapsto \mathbb{R}$, define the supremum norm $\|h\|_{\mathcal{F}} = \sup_{f\in\mathcal{F}}|h(f)|$. For the term $B_{n,1}(f)$ in (6) we have

$$\begin{aligned}
\|B_{n,1}\|_{\mathcal{F}} &\leqslant \left|\sqrt{n}\mathbb{P}_n\left(\frac{1}{g_{\hat{\pi}_n}} - \frac{1}{g_{\pi_0}}\right)\left(\xi_{\hat{\Lambda}_n} - \xi_{\Lambda_0}\right)\right| \\
&\leqslant \sqrt{n}\max_{1\leqslant i\leqslant n}\left|\frac{1}{g_{\hat{\pi}_n}(D_i)} - \frac{1}{g_{\pi_0}(D_i)}\right|\sup_{t\in[0,\tau]}\left|\exp\{\hat{\Lambda}_n(t)\} - \exp\{\Lambda_0(t)\}\right|\tau \\
&\leqslant \max\left\{\frac{1}{\hat{\pi}_n\pi_0}, \frac{1}{(1-\hat{\pi}_n)(1-\pi_0)}\right\}\left|\sqrt{n}(\hat{\pi}_n - \pi_0)\right| \\
&\quad \times \sup_{t\in[0,\tau]}\left|\exp\{\hat{\Lambda}_n(t)\} - \exp\{\Lambda_0(t)\}\right|\tau.
\end{aligned}$$

The last inequality, along with the boundedness of $\max[(\hat{\pi}_n\pi_0)^{-1}, \{(1-\hat{\pi}_n)(1-\pi_0)\}^{-1}]$ for all n sufficiently large, as a result of assumption A3, the fact that $\sqrt{n}(\hat{\pi}_n - \pi_0) = O_p(1)$, as

a consequence of the central limit theorem, the fact that

$$\sup_{t\in[0,\tau]}\left|\exp\{\hat{\Lambda}_n(t)\}-\exp\{\Lambda_0(t)\}\right|=o_{as*}(1),$$

as a result of the strong uniform consistency of the Nelson–Aalen estimator of the cumulative hazard (guaranteed by conditions C1 and C4) and the continuous mapping theorem, and the boundedness of the length of the follow-up interval τ, lead to the conclusion that $\|B_{n,1}\|_{\mathcal{F}}=o_p(1)$.

The term $B_{n,2}(f)$ can be expressed as follows

$$B_{n,2}(f)=\sqrt{n}(\mathbb{P}_n-P)\frac{\xi_{\hat{\Lambda}_n}-\xi_{\Lambda_0}}{g_{\pi_0}}u_f+\sqrt{n}P\frac{\xi_{\hat{\Lambda}_n}-\xi_{\Lambda_0}}{g_{\pi_0}}u_f. \tag{7}$$

The class of functions

$$\mathcal{F}=\{f(\cdot)=\beta_0+\langle\beta,\cdot\rangle:\beta_0\in\mathbb{R},\beta\in\mathbb{R}^p\}$$

is a Vapnik-Červonenkis (VC) class by Lemma 9.6 in Kosorok (2008). This along with lemma 9.9 and theorem 9.2 in Kosorok (2008) imply that the class $\{u_f:f\in\mathcal{F}\}$ has bounded uniform entropy integral. In addition, the latter class can be easily argued to be pointwise measurable and, therefore, this class is P-Donsker. By conditions C2 and C4 and Lemma 1, the fact that the class $\{\xi_\Lambda:\Lambda\in\mathcal{L}_\delta\}$ is uniformly bounded by $\tau\exp\{\Lambda_0(\tau)+\delta\}$, assumption A3, and the fact that products of uniformly bounded P-Donsker classes are also P-Donsker, it follows that the class

$$\left\{\frac{\xi_\Lambda-\xi_{\Lambda_0}}{g_{\pi_0}}u_f:\Lambda\in\mathcal{L}_\delta,f\in\mathcal{F}\right\}$$

is P-Donsker for some $\delta>0$. Also, by assumption A3, we have

$$\begin{aligned}\sup_{f\in\mathcal{F}}P\left(\frac{\xi_\Lambda-\xi_{\Lambda_0}}{g_{\pi_0}}u_f\right)^2 &\leqslant \left\{\frac{\tau\|\exp(\Lambda)-\exp(\Lambda_0)\|_{[0,\tau]}}{c_1\wedge(1-c_2)}\right\}^2\\ &\leqslant \left[\frac{\tau\exp\{\Lambda_0(\tau)\}\{\exp(\|\Lambda-\Lambda_0\|_{[0,\tau]})-1\}}{c_1\wedge(1-c_2)}\right]^2\to 0\end{aligned}$$

as $\|\Lambda-\Lambda_0\|_{[0,\tau]}\to 0$. The last two results along with the uniform consistency of the Nelson–Aalen estimator, guaranteed by conditions C1 and C4, and arguments similar to those used

in the proof of Lemma 3.3.5 in van der Vaart and Wellner (1996) lead to the conclusion that

$$\left\| \sqrt{n}(\mathbb{P}_n - P)\frac{\xi_{\hat{\Lambda}_n} - \xi_{\Lambda_0}}{g_{\pi_0}} u_f \right\|_{\mathcal{F}} = o_p(1).$$

For the second term in the right side of (7), it can be shown that the map $\Lambda \mapsto P g_{\pi_0}^{-1} \xi_\Lambda u_f$ is Hadamard differentiable at Λ_0 with derivative

$$\eta'_{\Lambda_0,f}(h) = P g_{\pi_0}^{-1} u_f \int_0^\tau Y(t) I(C \geqslant T \wedge t) \exp\{\Lambda_0(\tilde{T} \wedge t)\} h(\tilde{T} \wedge t) \mathrm{d}m(t).$$

This, along with the functional delta method (Theorem 3.9.4 in van der Vaart and Wellner, 1996) and the fact that

$$\sqrt{n}\{\hat{\Lambda}_n(t) - \Lambda_0(t)\} = \frac{1}{\sqrt{n}} \sum_{i=1}^n \gamma_i(t) + o_p(1), \qquad t \in [0, \tau],$$

where

$$\gamma_i(t) = \int_0^t \frac{\mathrm{d}N_i(s)}{PY(s)} - \int_0^t \frac{Y_i(s)}{PY(s)} \mathrm{d}\Lambda_0(s),$$

lead to the conclusion that

$$\begin{aligned} \sqrt{n} P \frac{\xi_{\hat{\Lambda}_n} - \xi_{\Lambda_0}}{g_{\pi_0}} u_f &= \frac{1}{\sqrt{n}} \sum_{i=1}^n \eta'_{\Lambda_0,f}(\gamma_i) + o_p(1) \\ &= \frac{1}{\sqrt{n}} \sum_{i=1}^n P g_{\pi_0}^{-1} u_f \int_0^\tau Y(t) I(C \geqslant T \wedge t) \exp\{\Lambda_0(\tilde{T} \wedge t)\} \gamma_i(\tilde{T} \wedge t) dm(t) \\ &\quad + o_p(1), \end{aligned}$$

where γ_i is considered fixed under the expectation operator P. Therefore,

$$B_{n,2}(f) = \frac{1}{\sqrt{n}} \sum_{i=1}^n \eta'_{\Lambda_0,f}(\gamma_i) + o_p(1) + \epsilon_{n,1}(f)$$

where

$$\epsilon_{n,1}(f) = \sqrt{n}(\mathbb{P}_n - P) \frac{\xi_{\hat{\Lambda}_n} - \xi_{\Lambda_0}}{g_{\pi_0}} u_f$$

with $\|\epsilon_{n,1}\|_{\mathcal{F}} = o_p(1)$.

For the term $B_{n,3}(f)$, we have

$$\begin{aligned} B_{n,3}(f) &= \sqrt{n}\mathbb{P}_n\xi_{\Lambda_0}\left(\frac{1}{g_{\hat{\pi}_n}}-\frac{1}{g_{\pi_0}}\right)u_f \\ &= -\left(\mathbb{P}_n\xi_{\Lambda_0}\frac{A}{g^2_{\pi^*_n}}u_f\right)\sqrt{n}(\hat{\pi}_n-\pi_0) \\ &= -\left(\mathbb{P}_n\xi_{\Lambda_0}\frac{A}{g^2_{\pi^*_n}}u_f - P\xi_{\Lambda_0}\frac{A}{g^2_{\pi_0}}u_f\right)\sqrt{n}(\hat{\pi}_n-\pi_0) \\ &\quad -\left(P\xi_{\Lambda_0}\frac{A}{g^2_{\pi_0}}u_f\right)\sqrt{n}(\hat{\pi}_n-\pi_0), \end{aligned} \tag{8}$$

where $|\pi^*_n-\pi_0| \leqslant |\hat{\pi}_n-\pi_0|$. For the first term in the right side of (8) we have

$$\begin{aligned} \sup_{f\in\mathcal{F}}\left|\mathbb{P}_n\xi_{\Lambda_0}\frac{A}{g^2_{\pi^*_n}}u_f - P\xi_{\Lambda_0}\frac{A}{g^2_{\pi_0}}u_f\right| &\leqslant \sup_{f\in\mathcal{F}}\left|\mathbb{P}_n\xi_{\Lambda_0}u_F A\left(\frac{1}{g^2_{\pi^*_n}}-\frac{1}{g^2_{\pi_0}}\right)\right| \\ &\quad +\sup_{f\in\mathcal{F}}\left|(\mathbb{P}_n-P)\xi_{\Lambda_0}\frac{A}{g^2_{\pi_0}}u_f\right| \\ &\leqslant \max\left\{\frac{1}{\pi^{*2}_n}-\frac{1}{\pi^2_0}, \frac{1}{(1-\pi^*_n)^2}-\frac{1}{(1-\pi_0)^2}\right\}\tau\exp\{\Lambda_0(\tau)\} \\ &\quad +\sup_{f\in\mathcal{F}}\left|(\mathbb{P}_n-P)\xi_{\Lambda_0}\frac{A}{g^2_{\pi_0}}u_f\right| \end{aligned}$$

The first term in the right side of the above inequality is $o_{as}(1)$ by assumption A3, the fact that $|\pi^*_n-\pi_0| \leqslant |\hat{\pi}_n-\pi_0|$, the strong law of large numbers, the continuous mapping theorem, and condition C4, which guarantees the finiteness of $\Lambda_0(\tau)$. The second term in the right side of the last inequaltity is $o_{as*}(1)$ as a consequence of the P-Donsker property of the class

$$\left\{\xi_{\Lambda_0}\frac{A}{g^2_{\pi_0}}u_f : f\in\mathcal{F}\right\},$$

which follows from similar arguments to those used in the analysis of the term $B_{n,2}(f)$ above, since this property implies that the latter class is also P-Glivenko–Cantelli. Therefore, using the last inequality gives

$$\sup_{f\in\mathcal{F}}\left|\mathbb{P}_n\xi_{\Lambda_0}\frac{A}{g^2_{\pi^*_n}}u_f - P\xi_{\Lambda_0}\frac{A}{g^2_{\pi_0}}u_f\right| = o_{as*}(1).$$

Consequently, given that $\sqrt{n}(\hat{\pi}_n-\pi_0)=O_p(1)$ by the central limit theorem, it follows from

(8) that

$$B_{n,3}(f) = -\left(P\xi_{\Lambda_0}\frac{A}{g^2_{\pi_0}}u_f\right)\sqrt{n}(\hat{\pi}_n - \pi_0) + \epsilon_{n,2}(f),$$

where

$$\epsilon_{n,2}(f) = -\left(\mathbb{P}_n\xi_{\Lambda_0}\frac{A}{g^2_{\pi^*_n}}u_f - P\xi_{\Lambda_0}\frac{A}{g^2_{\pi_0}}u_f\right)\sqrt{n}(\hat{\pi}_n - \pi_0),$$

with $\|\epsilon_{n,2}\|_{\mathcal{F}} = o_{as*}(1)O_p(1) = o_p(1)$.

Taking all the pieces together, it follows from (6) that

$$\begin{aligned}\sqrt{n}\left\{\hat{\mathcal{V}}_n(\text{sgn}(f)) - \mathcal{V}(\text{sgn}(f))\right\} &= \frac{1}{\sqrt{n}}\sum_{i=1}^{n}\Bigg[\eta'_{\Lambda_0,f}(\gamma_i) - \left(P\xi_{\Lambda_0}\frac{A}{g^2_{\pi_0}}u_f\right)\{I(A_i = 1) - \pi_0\} \\ &\quad + \left\{\frac{\xi_{\Lambda_0}(D_i)}{g_{\pi_0}(D_i)}u_f(D_i) - \mathcal{V}(\text{sgn}(f))\right\}\Bigg] + \epsilon_n(f) \\ &= \frac{1}{\sqrt{n}}\sum_{i=1}^{n}\psi_i(f) + \epsilon_n(f),\end{aligned}$$

where $\epsilon_n(f) = B_{n,1}(f) + \epsilon_{n,1}(f) + \epsilon_{n,2}(f) + o_p(1)$ with $\|\epsilon_n\|_{\mathcal{F}} = o_p(1)$. Finally, the class of functions $\{\psi(f) : f \in \mathcal{F}\}$ is P-Donsker as a consequence of the P-Donsker property of the class $\{u_f : f \in \mathcal{F}\}$ as argued above, Lemma 15.10 in Kosorok (2008), and the fact that sums of P-Donsker classes which are multiplied by random variables with finite second moments are also P-Donsker.

A.4 *Proof of Theorem 4*

Let $\hat{f}_n(\cdot) = \hat{\beta}_{n,0} + \langle\hat{\beta}_{n,1}, \cdot\rangle$, $\hat{\beta}_n = (\hat{\beta}_{n,0}, \hat{\beta}'_{n,1})'$ and $\tilde{\beta} = (\tilde{\beta}_0, \tilde{\beta}'_1)'$. Then, by Theorem 2, condition C5, and similar arguments to those used in Jiang et al. (2008), it follows that $\tilde{\beta}$ is unique and $\|\hat{\beta}_n - \tilde{\beta}\|_2 = o_p(1)$, where $\|\cdot\|_2$ is the Euclidean norm. Next, using the notation $f_\beta(\cdot) = \beta_0 + \langle\beta'_1, \cdot\rangle$, $\beta = (\beta_0, \beta'_1)'$, Thorem 3 guarantees that the class

$$\left\{\psi(f_\beta) - \psi(f_{\tilde{\beta}}) : \|\beta - \tilde{\beta}\|_2 < \delta\right\}$$

is P-Donsker for any $\delta > 0$. Moreover, by the assumption that $P(f_{\tilde{\beta}}(Z) = 0) = 0$ which implies the continuity of the map $\beta \mapsto I(af_\beta(z) \geqslant 0)$ at $\tilde{\beta}$ for almost all $z \in \mathcal{Z}$, it follows

that

$$P\{\psi(f_\beta) - \psi(f_{\tilde\beta})\}^2 \to 0 \ \text{ as } \ \beta \to \tilde\beta.$$

Next, by Theorem 3 and arguments similar to those used in the proof of Lemma 3.3.5 in van der Vaart and Wellner (1996) it follows that

$$\left|\sqrt{n}\left\{\hat{\mathcal{V}}_n(\text{sgn}(f_{\hat\beta_n})) - \mathcal{V}(\text{sgn}(f_{\hat\beta_n}))\right\} - \sqrt{n}\left\{\hat{\mathcal{V}}_n(\text{sgn}(f_{\tilde\beta})) - \mathcal{V}(\text{sgn}(f_{\tilde\beta}))\right\}\right| = o_p(1),$$

for any preference weight w that satisfies the requirements of Section 2 in the main text. Finally, the conclusion of Theorem 4 follows from the last result and the fact that the set $\mathcal{W}$ of preference weights is finite.

Web Appendix B. Influence Functions

Using traditional (instead of empirical process) notation, the influence functions $\psi_i(f)$ have the form

$$\begin{aligned}\psi_{i,w}(f) &= \int_0^\tau \frac{Y_{i,w}(t)I(C_i \geqslant T_i\wedge t)I[A_i = \text{sgn}\{f(Z_i)\}]}{\exp\{-\Lambda_0(\tilde T_i\wedge t)\}\{A_i\pi_0 + (1-A_i)/2\}}\text{d}m(t) - \mathcal{V}_w(\text{sgn}(f)) \\ &\quad - E\left\{A\int_0^\tau \frac{Y_w(t)I(C\geqslant T\wedge t)I[A = \text{sgn}\{f(Z)\}]}{\exp\{-\Lambda_0(\tilde T\wedge t)\}\{A\pi_0 + (1-A)/2\}^2}\text{d}m(t)\right\}\{I(A_i=1) - \pi_0\} \\ &\quad + \eta'_{\Lambda_0,f,w}(\gamma_i), \quad i = 1,\ldots,n, \;\; w\in\mathcal{W}, \;\; f\in\mathcal{F},\end{aligned}$$

where

$$\eta'_{\Lambda_0,f,w}(h) = E\left\{\int_0^\tau \frac{Y_w(t)I(C\geqslant T\wedge t)I[A = \text{sgn}\{f(Z)\}]}{\exp\{-\Lambda_0(\tilde T\wedge t)\}\{A\pi_0 + (1-A)/2\}} h(\tilde T\wedge t)\text{d}m(t)\right\},$$

for h in the space $D[0,\tau]$ of right continuous functions on $[0,\tau]$ with left hand limits and

$$\gamma_i(t) = \int_0^t \frac{dN_i(s)}{EY(s)} - \int_0^t \frac{Y_i(s)}{EY(s)}\text{d}\Lambda_0(s), \qquad i = 1,\ldots,n, \;\; t\in[0,\tau].$$

The empirical versions of the influence functions are

$$\begin{aligned}\hat\psi_{i,w}(f) &= \int_0^\tau \frac{Y_{i,w}(t)I(C_i > T_i\wedge t)I[A_i = \text{sgn}\{f(Z_i)\}]}{\exp\{-\hat\Lambda_n(\tilde T_i\wedge t)\}\{A_i\hat\pi_n + (1-A_i)/2\}}\text{d}m(t) - \hat{\mathcal{V}}_{n,w}(\text{sgn}(f)) \\ &\quad - \left\{\frac{1}{n}\sum_{j=1}^n A_j\int_0^\tau \frac{Y_{j,w}(t)I(C_j > T_j\wedge t)I[A_j = \text{sgn}\{f(Z_j)\}]}{\exp\{-\hat\Lambda_n(\tilde T_j\wedge t)\}\{A_j\hat\pi_n + (1-A_j)/2\}^2}\text{d}m(t)\right\}\{I(A_i=1) - \hat\pi_n\} \\ &\quad + \hat\eta'_{\hat\Lambda_n,f,w}(\hat\gamma_i), \quad i = 1,\ldots,n, \;\; w\in\mathcal{W}, \;\; f\in\mathcal{F},\end{aligned}$$

where

$$\hat{\eta}'_{\hat{\Lambda}_n,f,w}(h) = \frac{1}{n}\sum_{i=1}^{n}\left\{\int_0^{\tau}\frac{Y_{i,w}(t)I(C_i > T_i \wedge t)I[A_i = \text{sgn}\{f(Z_i)\}]}{\exp\{-\hat{\Lambda}_n(\tilde{T}_i \wedge t)\}\{A_i\hat{\pi}_n + (1-A_i)/2\}}h(\tilde{T}_i \wedge t)\text{d}m(t)\right\},$$

for $h \in D[0,\tau]$, and

$$\hat{\gamma}_i(t) = \int_0^t \frac{\text{d}N_i(s)}{n^{-1}\sum_{j=1}^{n} Y_j(s)} - \int_0^t \frac{Y_i(s)}{n^{-1}\sum_{j=1}^{n} Y_j(s)}\text{d}\hat{\Lambda}_n(s), \qquad i = 1,\ldots,n, \quad t \in [0,\tau].$$

Web Appendix C. Additional Simulation Results

In this Web Appendix we provide additional simulation results. Before these results, we first provide some discussion on the equality $\text{sgn}(f^*_{w_1}) = \text{sgn}(f^*_{w_2}) = \text{sgn}(f^*_w)$, under the simulation setup in this manuscript. Even though it is straightforward to see that $\text{sgn}(f^*_{w_1}) = \text{sgn}(f^*_w)$, it is not immediately clear why $\text{sgn}(f^*_{w_2}) = \text{sgn}(f^*_w)$. Before providing details for the latter, we need to define the state occupation probabilities

$$P_j(t;a,z) = P(X(t) = j | A = a, Z = z), \qquad j = 1,2,3,$$

and the transition probabilities

$$P_{hj}(s,t;a,z) = P(X(t) = j | X(s) = h; A = a, Z = z), \qquad h = 1,2, \quad j = 1,2,3.$$

For the progressive illness-death model (also known as the illness-death model without recovery) which was considered in the simulations, we have that

$$P_j(t;a,z) = P_{1j}(0,t;a,z), \qquad j = 1,2, \tag{9}$$

since $P(X(0) = 1|A = a, Z = z) = 1$. For the progressive illness-death model we also have, by the transition probabilities given in example IV.4.3 in Andersen et al. (1993) and under the transition intensities $\alpha_{12}(A,Z)$, $\alpha_{13}(A,Z)$, and $\alpha_{23}(A,Z)$ considered in the simulation studies, that

$$P_{11}(0,t;A,Z) = \exp[-\{\alpha_{12}(A,Z) + \alpha_{13}(A,Z)\}t]$$

and

$$P_{12}(0,t;A,Z) = \alpha_{12}(A,Z)\int_0^t P_{11}(0,s;A,Z)P_{22}(s,t;A,Z)ds,$$

where $P_{22}(s,t;A,Z) = \exp\{-\alpha_{23}(A,Z)(t-s)\}$ (see example IV.4.3 in Andersen et al., 1993). Therefore, by (9), we have that

$$P_1(t;A,Z) = \exp[-\{\alpha_{12}(A,Z) + \alpha_{13}(A,Z)\}t]$$

and

$$\begin{aligned} P_2(t;A,Z) &= \alpha_{12}(A,Z)\int_0^t P_{11}(0,s;A,Z)P_{22}(s,t;A,Z)ds \\ &= \alpha_{12}(A,Z)\int_0^t e^{-\{\alpha_{12}(A,Z)+\alpha_{13}(A,Z)\}s-\alpha_{23}(A,Z)(t-s)}ds \\ &= \alpha_{12}(A,Z)e^{-\alpha_{23}(A,Z)t}\int_0^t e^{-\{\alpha_{12}(A,Z)+\alpha_{13}(A,Z)-\alpha_{23}(A,Z)\}s}ds \\ &= \alpha_{12}(A,Z)e^{-\alpha_{23}(A,Z)t}\frac{1-e^{-\{\alpha_{12}(A,Z)+\alpha_{13}(A,Z)-\alpha_{23}(A,Z)\}t}}{\alpha_{12}(A,Z)+\alpha_{13}(A,Z)-\alpha_{23}(A,Z)}. \end{aligned}$$

The optimal decision function under the preference weight $w_2 = (1,1,0)'$ satisfies

$$\text{sgn}\{f^*_{w_2}(z)\} = \text{sgn}\left[\int_0^\tau \{P_1(t;1,z) + P_2(t;1,z)\}dt - \int_0^\tau \{P_1(t;-1,z) + P_2(t;-1,z)\}dt\right],$$

for all $z \in [-1,1]^2$, $\tau \in \{1,2,3\}$ (the τ values considered in the simulation studies), and the four scenarios considered, where $\int_0^\tau \{P_1(t;a,z) + P_2(t;a,z)\}dt$ is the expected time spent in the transient states 1 and 2 for $A = a$ and $Z = z$, by time τ. Defining

$$\gamma(A,Z) = \frac{\alpha_{12}(A,Z)}{\alpha_{12}(A,Z)+\alpha_{13}(A,Z)-\alpha_{23}(A,Z)},$$

it is straightforward to see that

$$\begin{aligned} P_1(t;A,Z) + P_2(t;A,Z) &= e^{-\{\alpha_{12}(A,Z)+\alpha_{13}(A,Z)\}t} + \gamma(A,Z)\left[e^{-\alpha_{23}(A,Z)t} - e^{-\{\alpha_{12}(A,Z)+\alpha_{13}(A,Z)\}t}\right] \\ &= \{1-\gamma(A,Z)\}e^{-\{\alpha_{12}(A,Z)+\alpha_{13}(A,Z)\}t} + \gamma(A,Z)e^{-\alpha_{23}(A,Z)t}, \end{aligned}$$

and thus

$$\begin{aligned} \int_0^\tau \{P_1(t;A,Z) + P_2(t;A,Z)\}dt &= \{1-\gamma(A,Z)\}\frac{1-e^{-\{\alpha_{12}(A,Z)+\alpha_{13}(A,Z)\}\tau}}{\alpha_{12}(A,Z)+\alpha_{13}(A,Z)} \\ &\quad +\gamma(A,Z)\frac{1-e^{-\alpha_{23}(A,Z)\tau}}{\alpha_{23}(A,Z)}. \end{aligned}$$

Given the highly nonlinear relationship between $\int_0^\tau \{P_1(t;A,Z) + P_2(t;A,Z)\}dt$ and $f_w^*(Z)$, showing that

$$\text{sgn}\{f_w^*(Z)\} = \text{sgn}\left[\int_0^\tau \{P_1(t;1,Z) + P_2(t;1,Z)\}dt - \int_0^\tau \{P_1(t;-1,Z) + P_2(t;-1,Z)\}dt\right], \tag{10}$$

for all Z is difficult. Equality (10) was shown numerically by simulating 100,000 vectors Z and confirming (10) for each simulated Z, $\tau \in \{1,2,3\}$, and simulation scenario $\in \{1,2,3,4\}$. Thus, we numerically shown that $\text{sgn}\{f_{w_2}^*(Z)\} = \text{sgn}\{f_w^*(Z)\}$ almost surely, under the simulating settings in this work. The intuitive interpretation of (10) is as follows. Under the specific choices for the transition intensities, if $f_w^*(z) > 0$ then provision of treatment $A = \text{sgn}\{f_w^*(z)\} = 1$ is associated with a reduction in the expected time spent in state 1, $\int_0^\tau P_1(t;1,z)dt$, which is outweighed by an increase in the expected time spent in state 2, $\int_0^\tau P_2(t;1,z)dt$. The same is true under treatment $A = -1$, if $f_w^*(z) < 0$.

C.1 *Additional simulation results under* $w = (0,1,0)'$ *(duration of tumor response)*

Simulation results evaluating the effect of selecting different values of τ in the analysis are illustrated in Figures 1–3.

[Figure 1 about here.]

[Figure 2 about here.]

[Figure 3 about here.]

Simulation results on the performance of the proposed ITR estimator when $\mathcal{F}$ is the RKHS with the Gaussian kernel with $\sigma = 1$ (less flexible kernel) and $\sigma = 5$ (more flexible kernel), for the duration of tumor response, are depicted in Figures 4–6. For comparison, these figures also illustrate the performance of the proposed method when $\mathcal{F}$ is the space of linear functions.

[Figure 4 about here.]

[Figure 5 about here.]

[Figure 6 about here.]

C.2 *Simulation results under* $w = (1, 1, 0)'$ *(progression-free survival time)*

Simulation results regarding the performance of the estimated ITR $\hat{d}_{n,w}$ for the progression-free survival time (i.e., $w = (1, 1, 0)'$) are depicted in Figures 7–9. For comparison, these plots also illustrate the performance of the inverse censoring weighted outcome weighted learning (ICO) and doubly robust outcome weighted learning (DR) methods by Zhao et al. (2015) for censored failure times.

[Figure 7 about here.]

[Figure 8 about here.]

[Figure 9 about here.]

The simulation results regarding the validity of the proposed inference methods for $\mathcal{V}_w(\hat{d}_{n,w})$ in terms of the progression-free survival time are summarized in Tables 1 and 2.

[Table 1 about here.]

[Table 2 about here.]

Simulation results on the performance of the proposed ITR estimator when $\mathcal{F}$ is the RKHS with the Gaussian kernel with $\sigma = 1$ (less flexible kernel) and $\sigma = 5$ (more flexible kernel), for the duration of tumor response, are depicted in Figures 10–12. For comparison, these figures also illustrate the performance of the proposed method when $\mathcal{F}$ is the space of linear functions.

[Figure 10 about here.]

[Figure 11 about here.]

[Figure 12 about here.]

Web Appendix D. Additional Results from the SPECTRUM Trial Analysis

The estimates of the treatment-specific cumulative transition intensities and state occupation probabilities based on the data from the SPECTRUM trial are depicted in Figures 13 and 14.

[Figure 13 about here.]

[Figure 14 about here.]

Web Appendix E. Relaxing the independent censoring assumption

As mentioned in the discussion of the main manuscript, a plausible relaxation of the independent censoring assumption (condition C1) is to allow censoring to depend on treatment A, since censoring will likely be higher among those receiving the treatment with the greater toxicity (Templeton et al., 2020). This can be trivially incorporated into the proposed methodology by simply replacing $\hat{\Lambda}_n(\tilde{T} \wedge t)$ in the proposed estimators with the nonparametric estimate of the conditional cumulative hazard of censoring given A, that is

$$\hat{\Lambda}_n(\tilde{T} \wedge t, A) = \hat{\Lambda}_{n,1}(\tilde{T} \wedge t)I(A = 1) + \hat{\Lambda}_{n,-1}(\tilde{T} \wedge t)I(A = -1),$$

where

$$\hat{\Lambda}_{n,a}(t) = \int_0^t \frac{\sum_{i=1}^n I(A_i = a)\mathrm{d}N_i(s)}{\sum_{i=1}^n I(A_i = a)Y_i(s)}, \quad a \in \{-1, 1\}, \quad t \in [0, \tau].$$

This estimator is uniformly consistent for the true conditional cumulative hazard of censoring given A

$$\Lambda_0(\tilde{T} \wedge t, A) = \Lambda_{0,1}(\tilde{T} \wedge t)I(A = 1) + \Lambda_{0,-1}(\tilde{T} \wedge t)I(A = -1),$$

where $\Lambda_{0,a}(t)$, $a \in \{-1, 1\}$, is the true cumulative hazard of censoring among those with $A = a$. In this case, all the theoretical poperties of the proposed estimators in the main manuscript still hold, with the exception that the influence functions $\psi_i(f)$ have the (slightly

different) form

$$\begin{aligned}\psi_{i,w}(f) &= \int_0^\tau \frac{Y_{i,w}(t)I(C_i \geqslant T_i \wedge t)I[A_i = \mathrm{sgn}\{f(Z_i)\}]}{\exp\{-\Lambda_0(\tilde{T}_i \wedge t, A_i)\}\{A_i\pi_0 + (1-A_i)/2\}}\mathrm{d}m(t) - \mathcal{V}_w(\mathrm{sgn}(f)) \\ &\quad -E\left\{A\int_0^\tau \frac{Y_w(t)I(C \geqslant T \wedge t)I[A = \mathrm{sgn}\{f(Z)\}]}{\exp\{-\Lambda_0(\tilde{T} \wedge t, A)\}\{A\pi_0 + (1-A)/2\}^2}\mathrm{d}m(t)\right\}\{I(A_i = 1) - \pi_0\} \\ &\quad +\eta'_{\Lambda_0,f,w}(\gamma_i), \quad i = 1,\ldots,n, \quad w \in \mathcal{W}, \quad f \in \mathcal{F},\end{aligned}$$

where

$$\mathcal{V}_w(d) = E\left(\left[\int_0^\tau \frac{Y_w(t)I(C \geqslant T \wedge t)}{\exp\{-\Lambda_0(\tilde{T} \wedge t, A)\}}\mathrm{d}m(t)\right]\frac{I(A = d(Z))}{A\pi_0 + (1-A)/2}\right),$$

$$\eta'_{\Lambda_0,f,w}(h) = E\left\{\int_0^\tau \frac{Y_w(t)I(C \geqslant T \wedge t)I[A = \mathrm{sgn}\{f(Z)\}]}{\exp\{-\Lambda_0(\tilde{T} \wedge t, A)\}\{A\pi_0 + (1-A)/2\}}h(\tilde{T} \wedge t, A)\mathrm{d}m(t)\right\},$$

for h in the space $D[0,\tau]$ of right continuous functions on $[0,\tau]$ with left hand limits and

$$\gamma_i(t,a) = \sum_{j\in\{-1,1\}} I(a = j)\left[\int_0^t \frac{I(A_i = j)dN_i(s)}{E\{I(A = j)Y(s)\}} - \int_0^t \frac{I(A_i = j)Y_i(s)}{E\{I(A = j)Y(s)\}}\mathrm{d}\Lambda_{0,a}(s)\right], \quad t \in [0,\tau],$$

for $i = 1,\ldots,n$. The corresponding empirical versions of the latter influence functions are

$$\begin{aligned}\hat{\psi}_{i,w}(f) &= \int_0^\tau \frac{Y_{i,w}(t)I(C_i > T_i \wedge t)I[A_i = \mathrm{sgn}\{f(Z_i)\}]}{\exp\{-\hat{\Lambda}_n(\tilde{T}_i \wedge t, A_i)\}\{A_i\hat{\pi}_n + (1-A_i)/2\}}\mathrm{d}m(t) - \hat{\mathcal{V}}_{n,w}(\mathrm{sgn}(f)) \\ &\quad -\left\{\frac{1}{n}\sum_{j=1}^n A_j\int_0^\tau \frac{Y_{j,w}(t)I(C_j > T_j \wedge t)I[A_j = \mathrm{sgn}\{f(Z_j)\}]}{\exp\{-\hat{\Lambda}_n(\tilde{T}_j \wedge t, A_j)\}\{A_j\hat{\pi}_n + (1-A_j)/2\}^2}\mathrm{d}m(t)\right\}\{I(A_i = 1) - \hat{\pi}_n\} \\ &\quad +\hat{\eta}'_{\hat{\Lambda}_n,f,w}(\hat{\gamma}_i), \quad i = 1,\ldots,n, \quad w \in \mathcal{W}, \quad f \in \mathcal{F},\end{aligned}$$

where

$$\hat{\mathcal{V}}_{n,w}(d) = \frac{1}{n}\sum_{i=1}^n\left(\left[\int_0^\tau \frac{Y_{i,w}(t)I(C_i \geqslant T_i \wedge t)}{\exp\{-\hat{\Lambda}_n(\tilde{T}_i \wedge t, A_i)\}}\mathrm{d}m(t)\right]\frac{I(A_i = d(Z_i))}{A_i\hat{\pi}_n + (1-A_i)/2}\right),$$

$$\hat{\eta}'_{\hat{\Lambda}_n,f,w}(h) = \frac{1}{n}\sum_{i=1}^n\left\{\int_0^\tau \frac{Y_{i,w}(t)I(C_i > T_i \wedge t)I[A_i = \mathrm{sgn}\{f(Z_i)\}]}{\exp\{-\hat{\Lambda}_n(\tilde{T}_i \wedge t, A_i)\}\{A_i\hat{\pi}_n + (1-A_i)/2\}}h(\tilde{T}_i \wedge t, A_i)\mathrm{d}m(t)\right\},$$

for $h \in D[0,\tau]$, and

$$\hat{\gamma}_i(t,a) = \sum_{j\in\{-1,1\}} I(a = j)\left[\int_0^t \frac{I(A_i = j)\mathrm{d}N_i(s)}{n^{-1}\sum_{l=1}^n I(A_l = j)Y_l(s)} - \int_0^t \frac{I(A_i = j)Y_i(s)}{n^{-1}\sum_{l=1}^n I(A_l = j)Y_l(s)}\mathrm{d}\hat{\Lambda}_{n,a}(s)\right],$$

for $i = 1,\ldots,n$ and $t \in [0,\tau]$.

A further relaxation of the independent censoring assumption is to allow censoring to depend on both A and Z. In this case, one can impose a semiparametric Cox model of the

form $\Lambda(t; A, Z) = \Lambda_0(t)\exp\{\theta'(A, Z')'\}$ for the right censoring time, and use the estimated conditional hazard in the proposed objective function and value function estimators. Provided that this model is correctly specified, the theoretical properties of the proposed method still hold, with the exception that $\gamma_i(t)$ in $\psi_{i,w}(f)$ (see Web Appendix B) is replaced by the influence function of $\sqrt{n}\{\hat{\Lambda}_n(t)\exp(\hat{\theta}_n'(a, z')') - \Lambda(t; a, z)\}$ under partial likelihood estimation.

References

Andersen, P. K., Borgan, O., Gill, R. D., and Keiding, N. (1993). *Statistical models based on counting processes.* Springer Science & Business Media.

Athreya, K. B. and Lahiri, S. N. (2006). *Measure theory and probability theory*, volume 19. Springer.

Hable, R. (2012). Asymptotic normality of support vector machine variants and other regularized kernel methods. *Journal of Multivariate Analysis* **106,** 92–117.

Jiang, B., Zhang, X., and Cai, T. (2008). Estimating the confidence interval for prediction errors of support vector machine classifiers. *Journal of Machine Learning Research* **9,** 521–540.

Kosorok, M. R. (2008). *Introduction to empirical processes and semiparametric inference.* Springer.

Templeton, A. J., Amir, E., and Tannock, I. F. (2020). Informative censoring–a neglected cause of bias in oncology trials. *Nature Reviews Clinical Oncology* **17,** 327–328.

Tsiatis, A. A., Davidian, M., Holloway, S. T., and Laber, E. B. (2019). *Dynamic treatment regimes: Statistical methods for precision medicine.* Chapman and Hall/CRC.

van der Vaart, A. W. and Wellner, J. A. (1996). *Weak convergence and empirical processes with applications to Statistics.* Springer.

Zhao, Y., Zeng, D., Rush, A. J., and Kosorok, M. R. (2012). Estimating individualized

treatment rules using outcome weighted learning. *Journal of the American Statistical Association* **107,** 1106–1118.

Zhao, Y.-Q., Zeng, D., Laber, E. B., Song, R., Yuan, M., and Kosorok, M. R. (2015). Doubly robust learning for estimating individualized treatment with censored data. *Biometrika* **102,** 151–168.

Zhou, X., Mayer-Hamblett, N., Khan, U., and Kosorok, M. R. (2017). Residual weighted learning for estimating individualized treatment rules. *Journal of the American Statistical Association* **112,** 169–187.

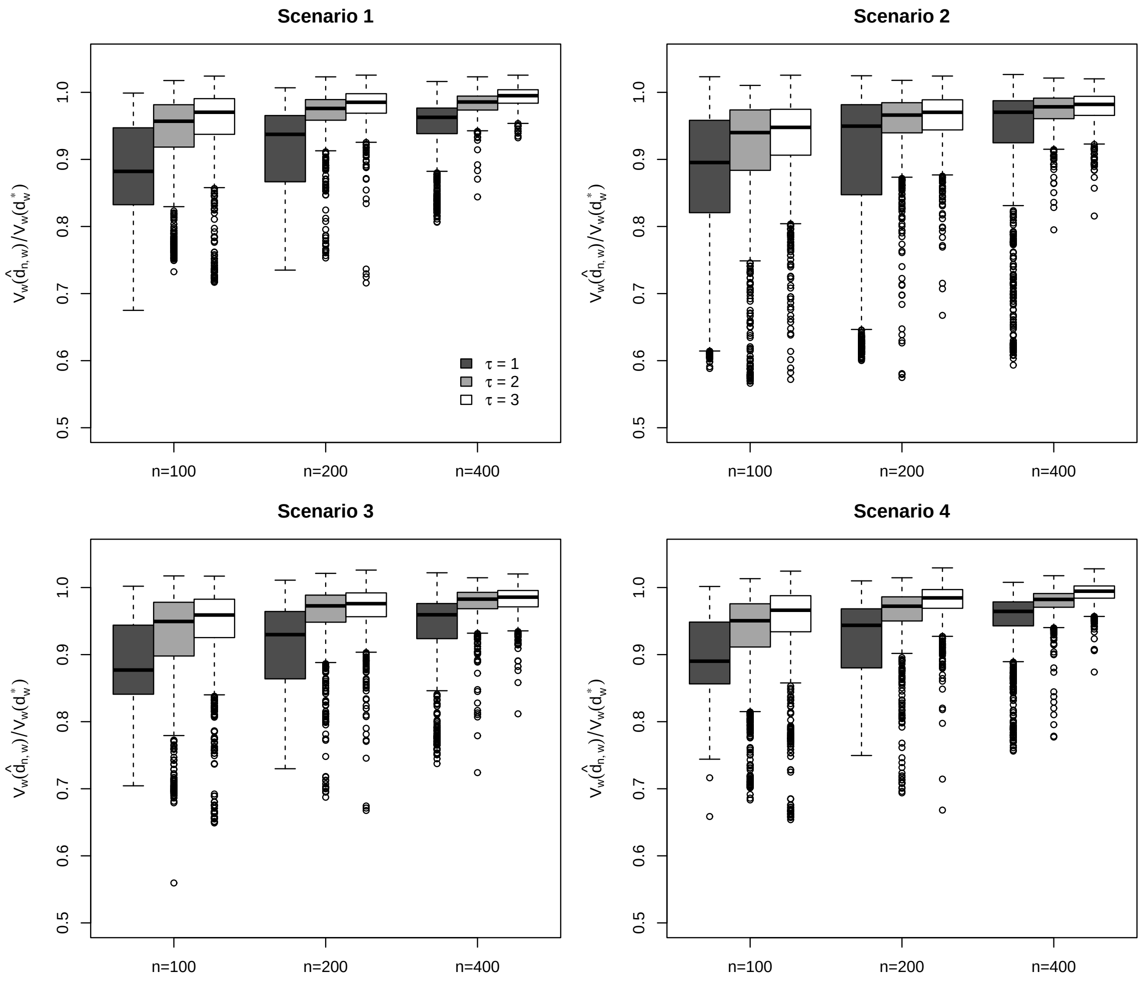

Figure 1. Simulation study: Performance of $\hat{d}_{n,w}$ for the duration of tumor response (i.e., $w = (0, 1, 0)'$), in terms of the estimated individualized treatment rule value ratio $\mathcal{V}_w(\hat{d}_{n,w})/\mathcal{V}_w(d_w^*)$ for different choices of the τ used in the analysis. The total length of the follow-up is equal to 3. Results under an average censoring rate of 28.4%.

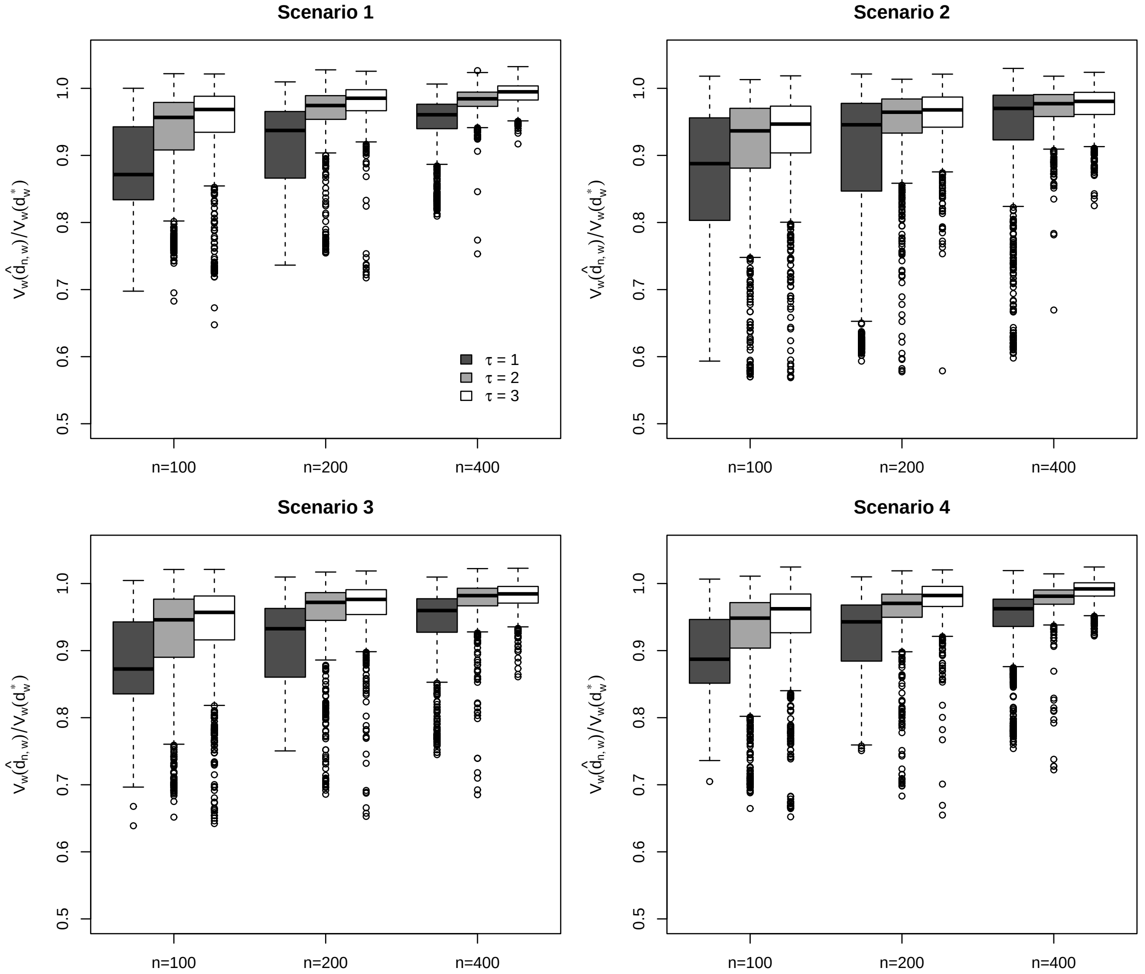

Figure 2. Simulation study: Performance of $\hat{d}_{n,w}$ for the duration of tumor response (i.e., $w = (0,1,0)'$), in terms of the estimated individualized treatment rule value ratio $\mathcal{V}_w(\hat{d}_{n,w})/\mathcal{V}_w(d_w^*)$ for different choices of the τ used in the analysis. The total length of the follow-up is equal to 3. Results under an average censoring rate of 42.8%.

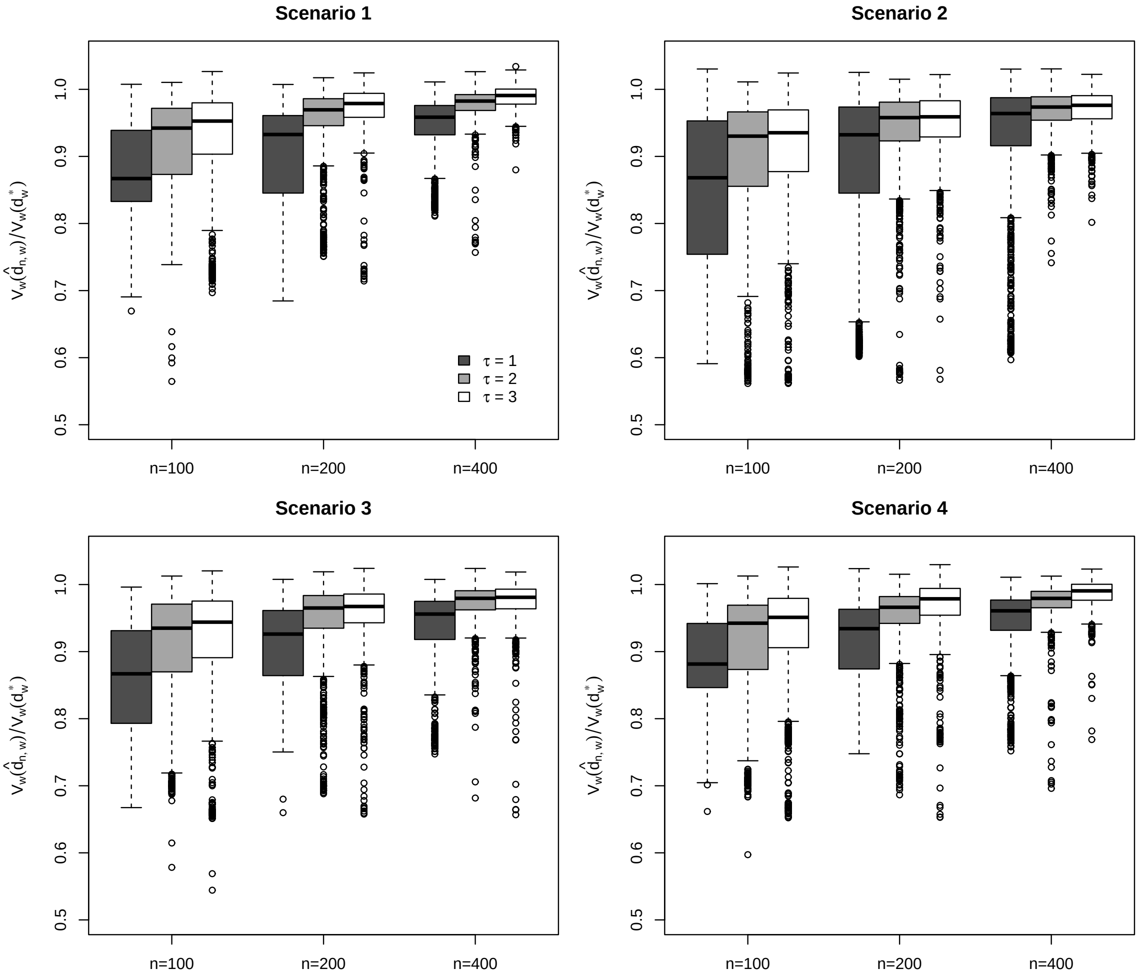

Figure 3. Simulation study: Performance of $\hat{d}_{n,w}$ for the duration of tumor response (i.e., $w = (0, 1, 0)'$), in terms of the estimated individualized treatment rule value ratio $\mathcal{V}_w(\hat{d}_{n,w})/\mathcal{V}_w(d^*_w)$ for different choices of the τ used in the analysis. The total length of the follow-up is equal to 3. Results under an average censoring rate of 59.5%.

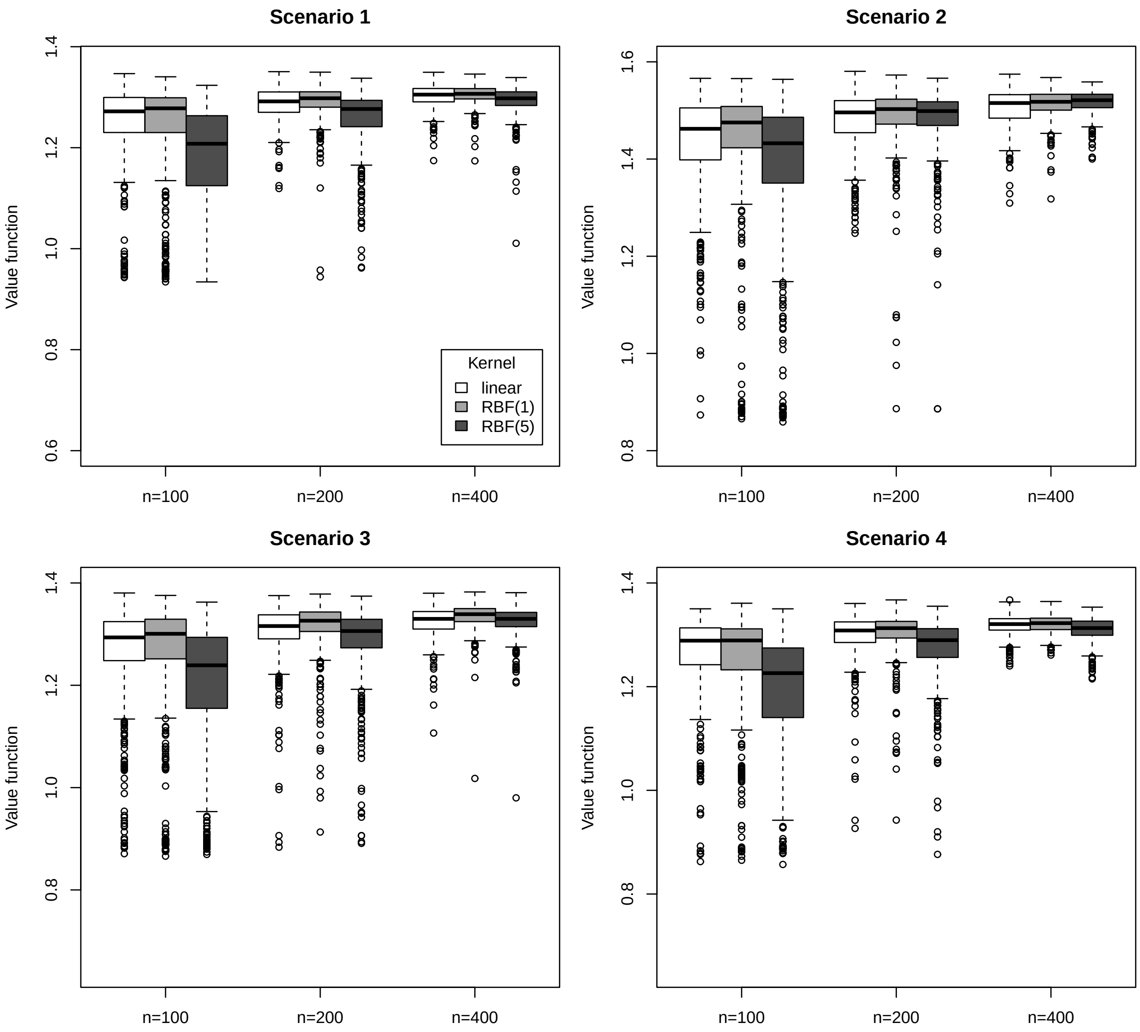

Figure 4. Simulation study: Value functions for the estimated individualized treatment rules for the duration of tumor response (i.e., $w = (0, 1, 0)'$), based on the proposed method with $\mathcal{F}$ being the class of linear functions or the RKHS with the Gaussian (also known as radial basis function) kernel with $\sigma = 1$ (RBF(1); less flexible kernel) and $\sigma = 5$ (RBF(5); more flexible kernel). Results under an average censoring rate of 28.4%.

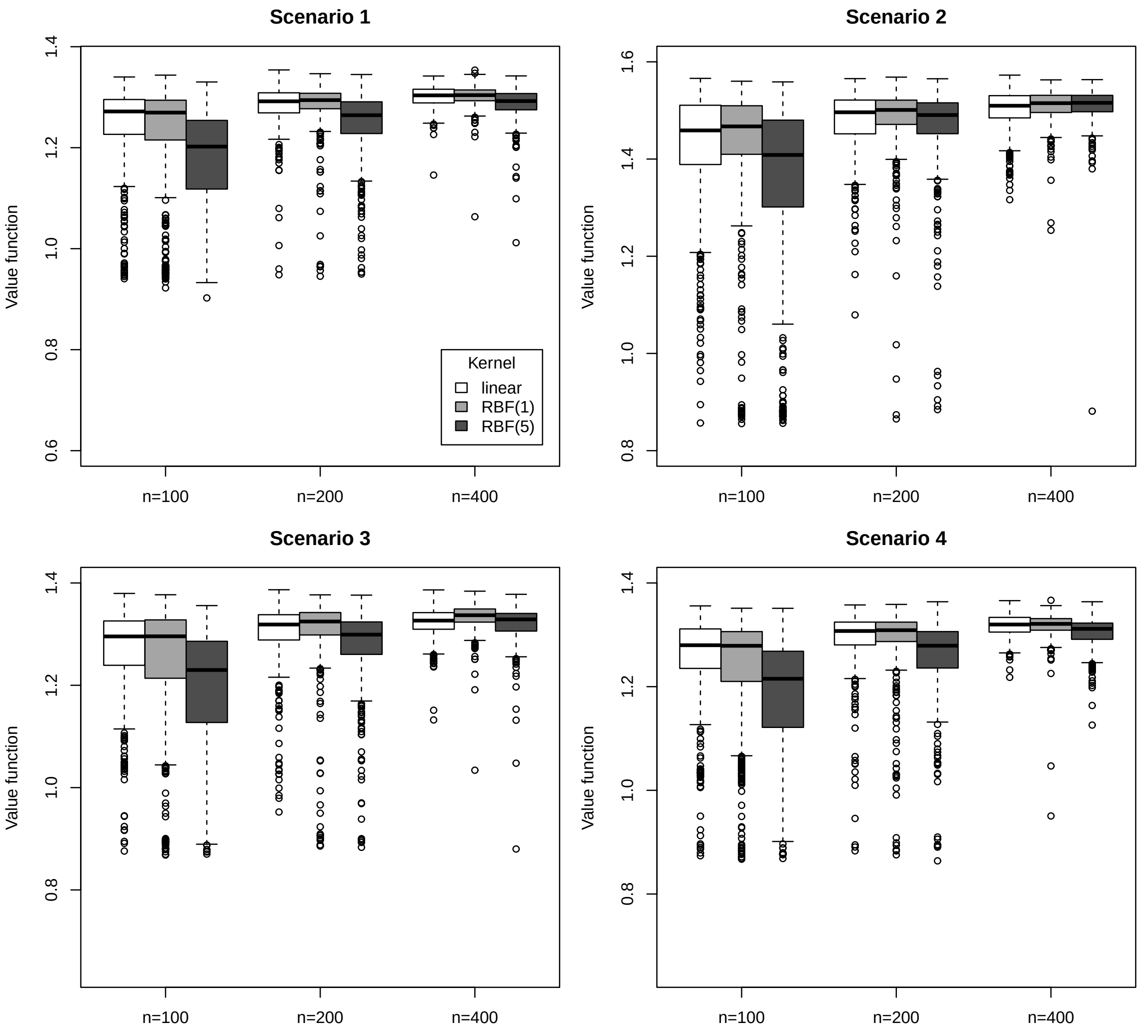

Figure 5. Simulation study: Value functions for the estimated individualized treatment rules for the duration of tumor response (i.e., $w = (0, 1, 0)'$), based on the proposed method with $\mathcal{F}$ being the class of linear functions or the RKHS with the Gaussian (also known as radial basis function) kernel with $\sigma = 1$ (RBF(1); less flexible kernel) and $\sigma = 5$ (RBF(5); more flexible kernel). Results under an average censoring rate of 42.8%.

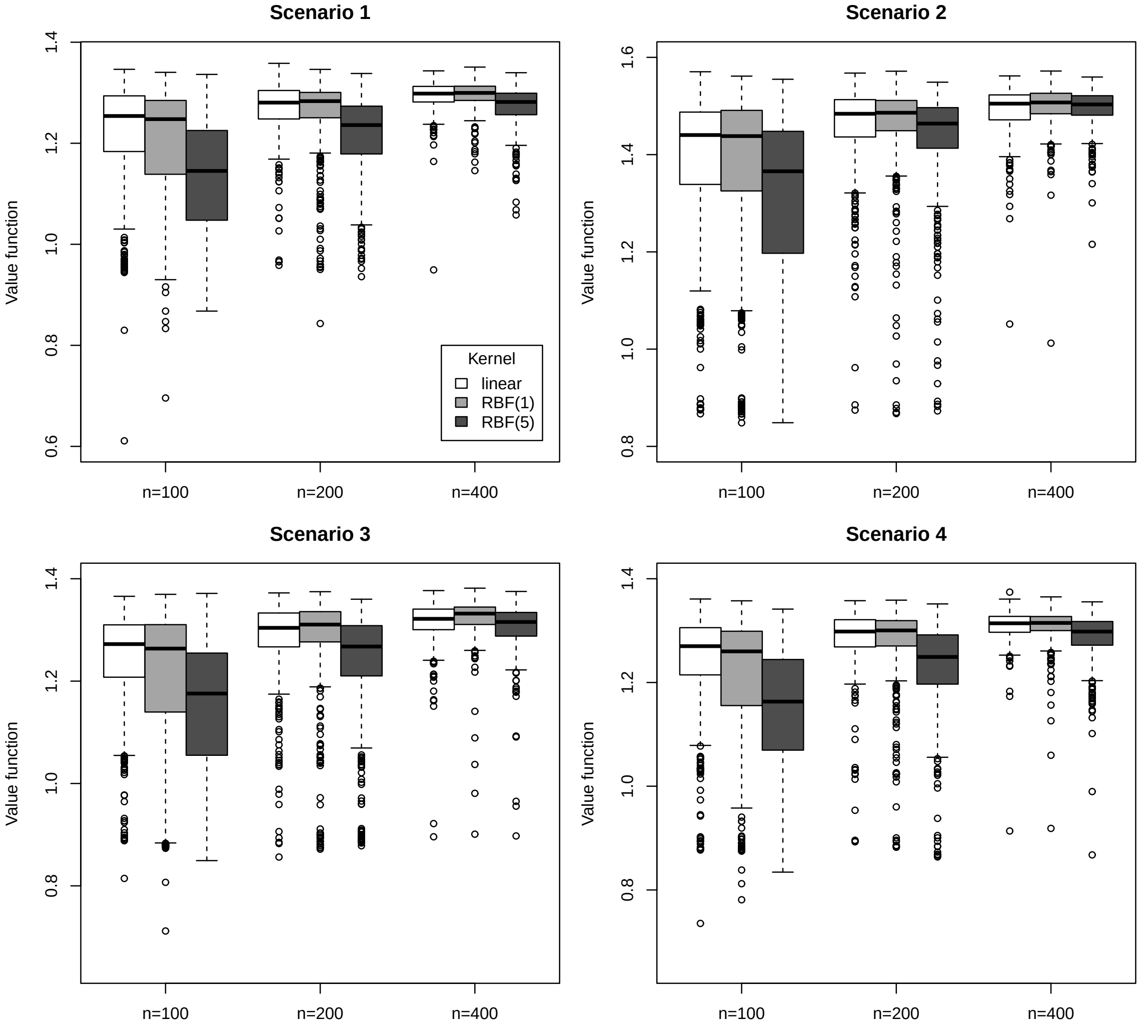

Figure 6. Simulation study: Value functions for the estimated individualized treatment rules for the duration of tumor response (i.e., $w = (0, 1, 0)'$), based on the proposed method with $\mathcal{F}$ being the class of linear functions or the RKHS with the Gaussian (also known as radial basis function) kernel with $\sigma = 1$ (RBF(1); less flexible kernel) and $\sigma = 5$ (RBF(5); more flexible kernel). Results under an average censoring rate of 59.5%.

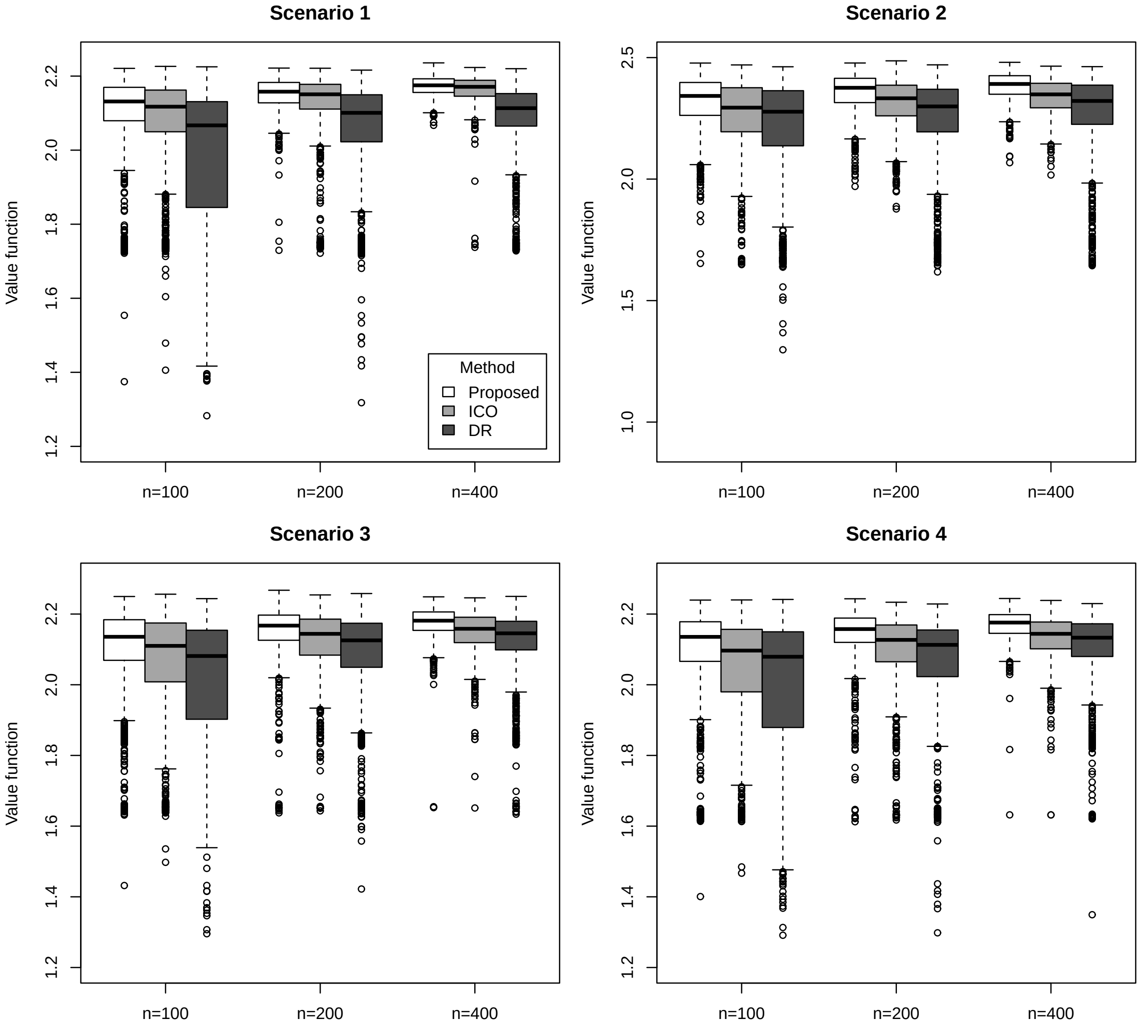

Figure 7. Simulation study: Value functions for the estimated individualized treatment rules based on the proposed method and the inverse censoring weighted outcome weighted learning (ICO) and doubly robust outcome weighted learning (DR) methods by Zhao et al. (2015) for the progression-free survival time (i.e., $w = (1, 1, 0)'$). Results under an average censoring rate of 28.4%.

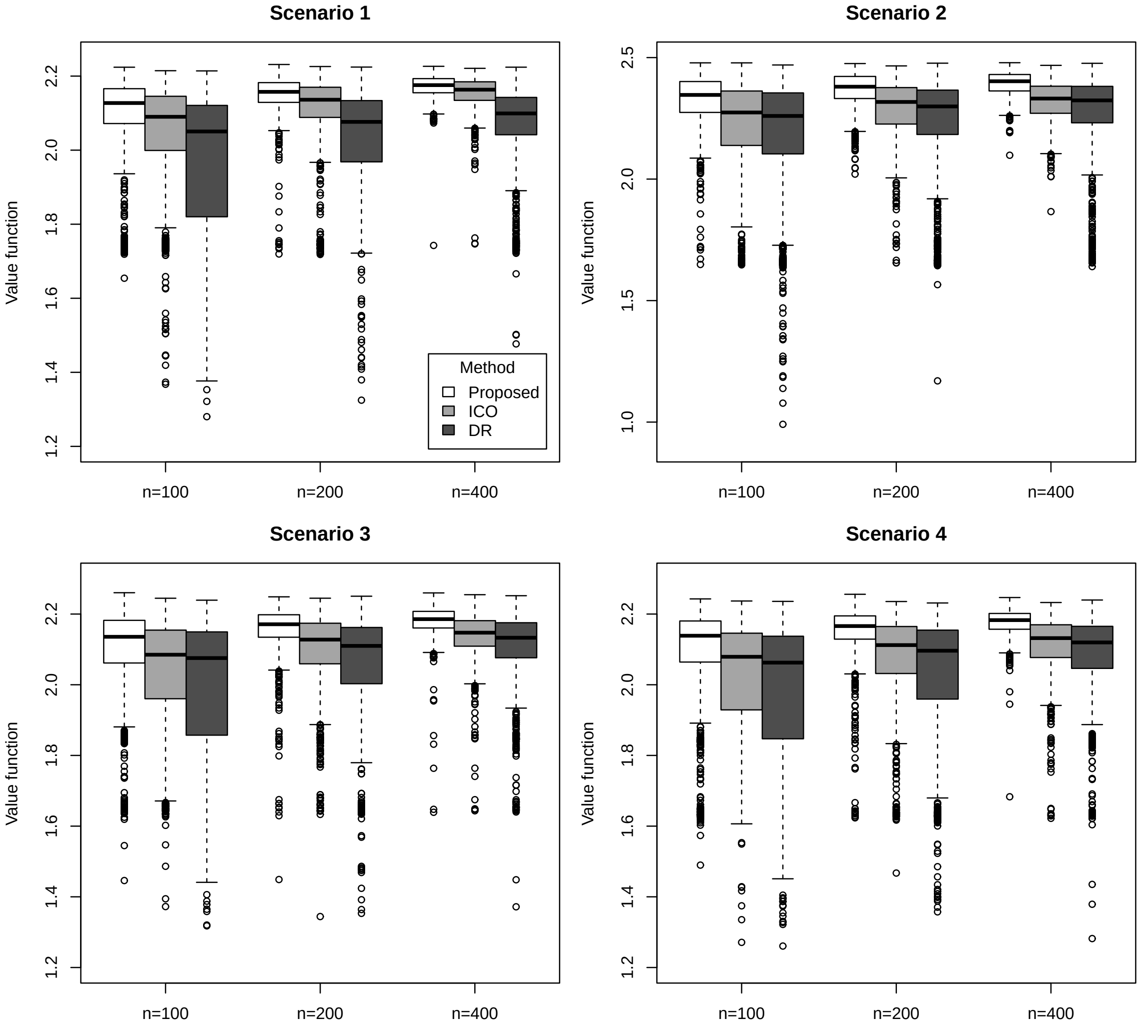

Figure 8. Simulation study: Value functions for the estimated individualized treatment rules based on the proposed method and the inverse censoring weighted outcome weighted learning (ICO) and doubly robust outcome weighted learning (DR) methods by Zhao et al. (2015) for the progression-free survival time (i.e., $w = (1, 1, 0)'$). Results under an average censoring rate of 42.8%.

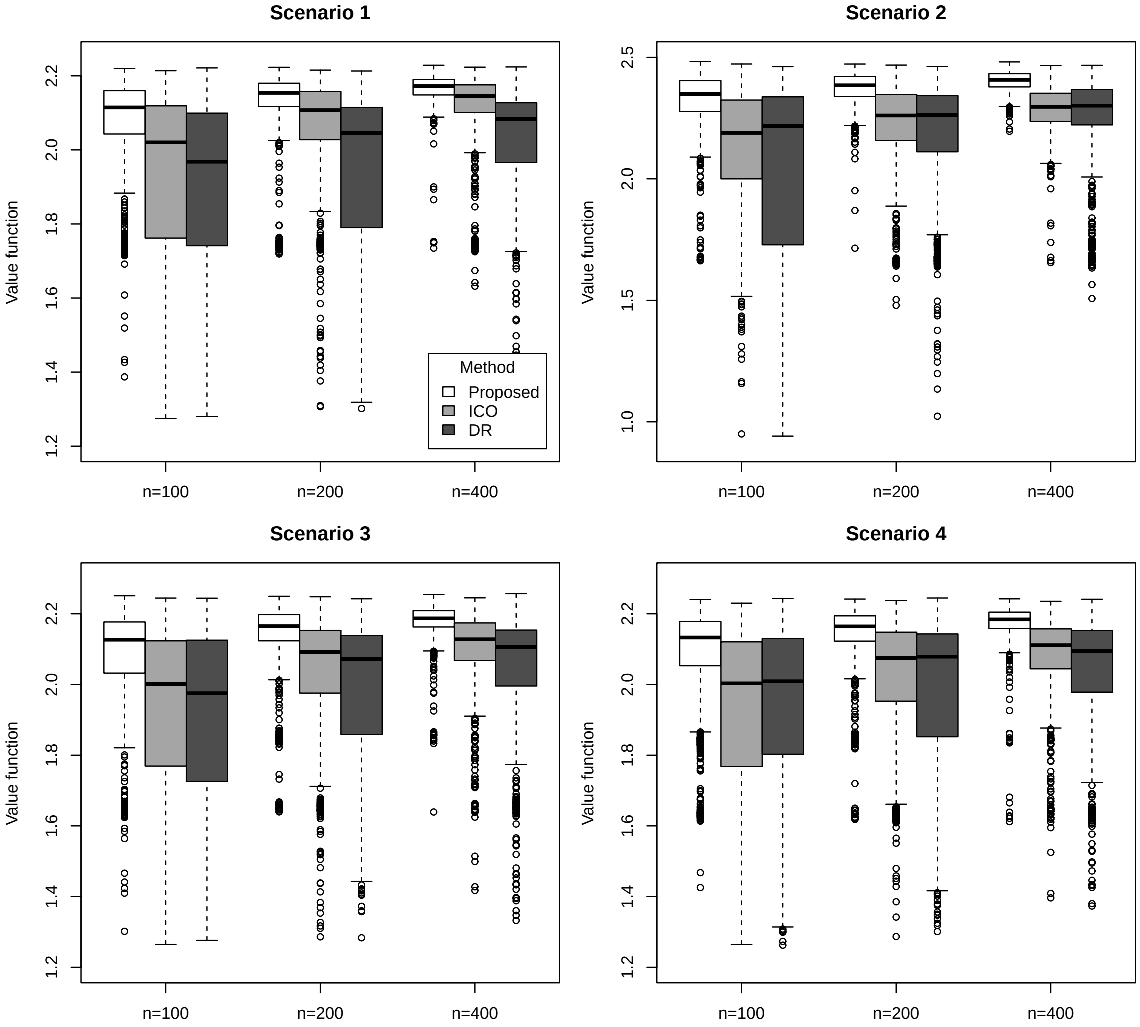

Figure 9. Simulation study: Value functions for the estimated individualized treatment rules based on the proposed method and the inverse censoring weighted outcome weighted learning (ICO) and doubly robust outcome weighted learning (DR) methods by Zhao et al. (2015) for the progression-free survival time (i.e., $w = (1, 1, 0)'$). Results under an average censoring rate of 59.5%.

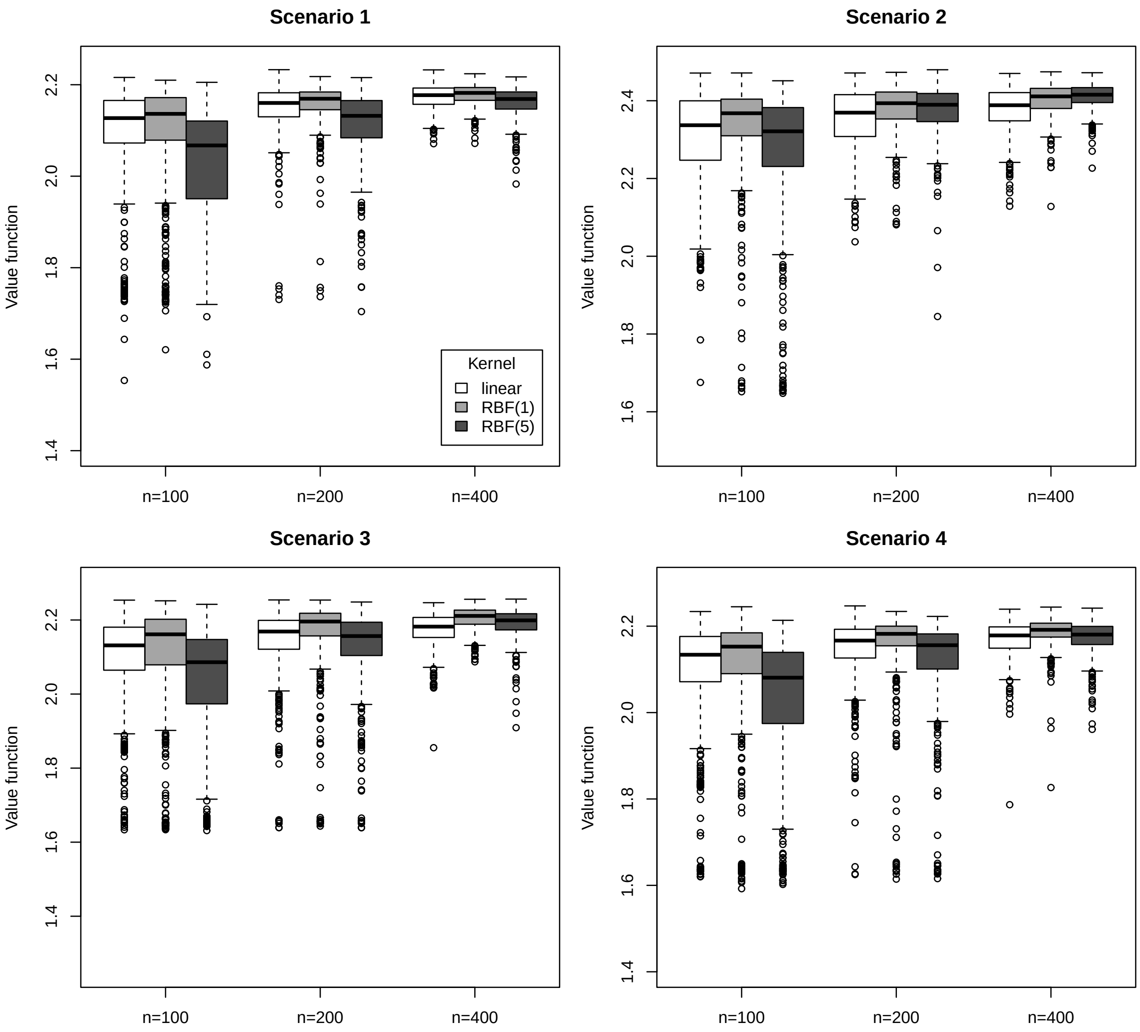

Figure 10. Simulation study: Value functions for the estimated individualized treatment rules for the progression-free survival time (i.e., $w = (1, 1, 0)'$), based on the proposed method with $\mathcal{F}$ being the class of linear functions or the RKHS with the Gaussian (also known as radial basis function) kernel with $\sigma = 1$ (RBF(1); less flexible kernel) and $\sigma = 5$ (RBF(5); more flexible kernel). Results under an average censoring rate of 28.4%.

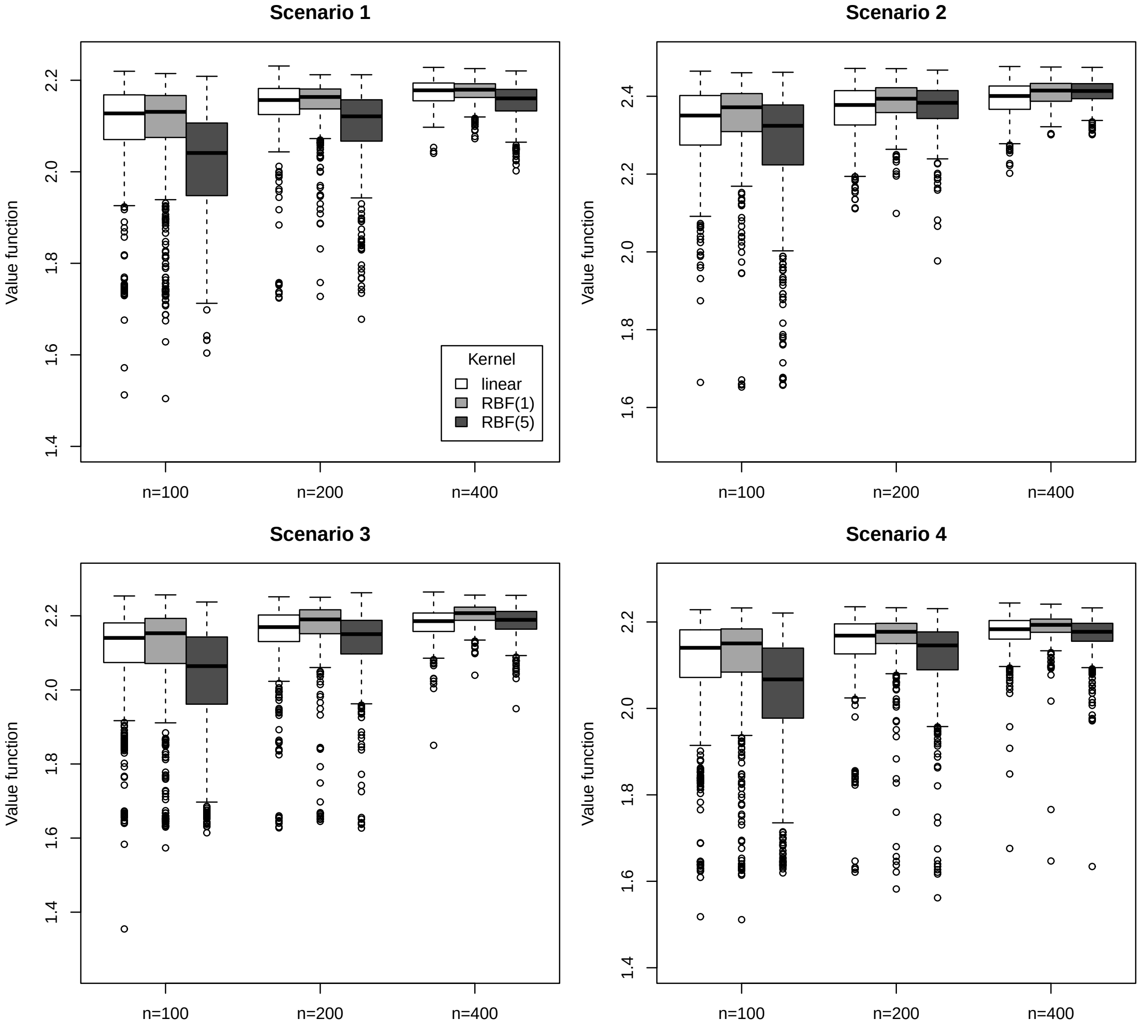

Figure 11. Simulation study: Value functions for the estimated individualized treatment rules for the progression-free survival time (i.e., $w = (1, 1, 0)'$), based on the proposed method with $\mathcal{F}$ being the class of linear functions or the RKHS with the Gaussian (also known as radial basis function) kernel with $\sigma = 1$ (RBF(1); less flexible kernel) and $\sigma = 5$ (RBF(5); more flexible kernel). Results under an average censoring rate of 42.8%.

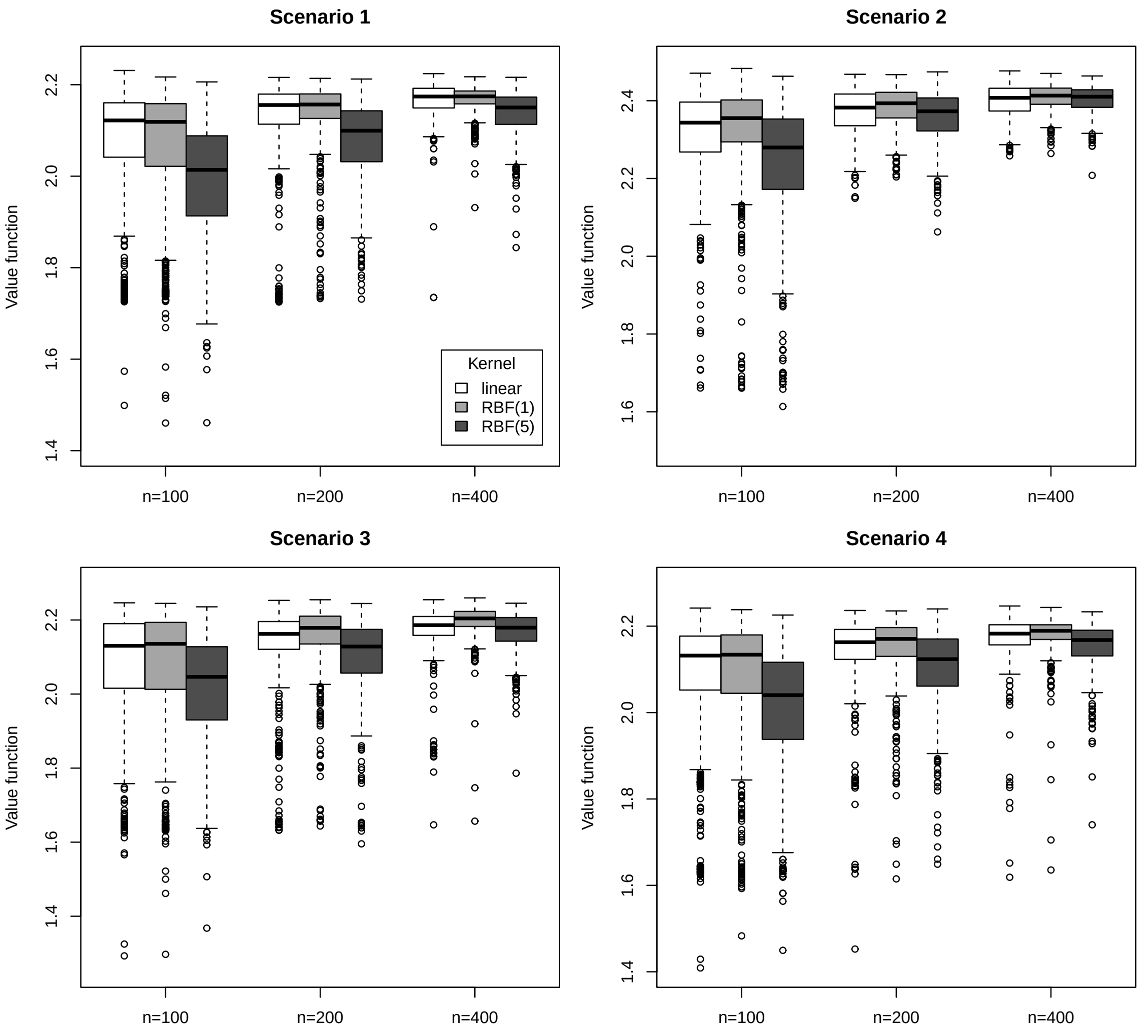

Figure 12. Simulation study: Value functions for the estimated individualized treatment rules for the progression-free survival time (i.e., $w = (1, 1, 0)'$), based on the proposed method with $\mathcal{F}$ being the class of linear functions or the RKHS with the Gaussian (also known as radial basis function) kernel with $\sigma = 1$ (RBF(1); less flexible kernel) and $\sigma = 5$ (RBF(5); more flexible kernel). Results under an average censoring rate of 59.5%.

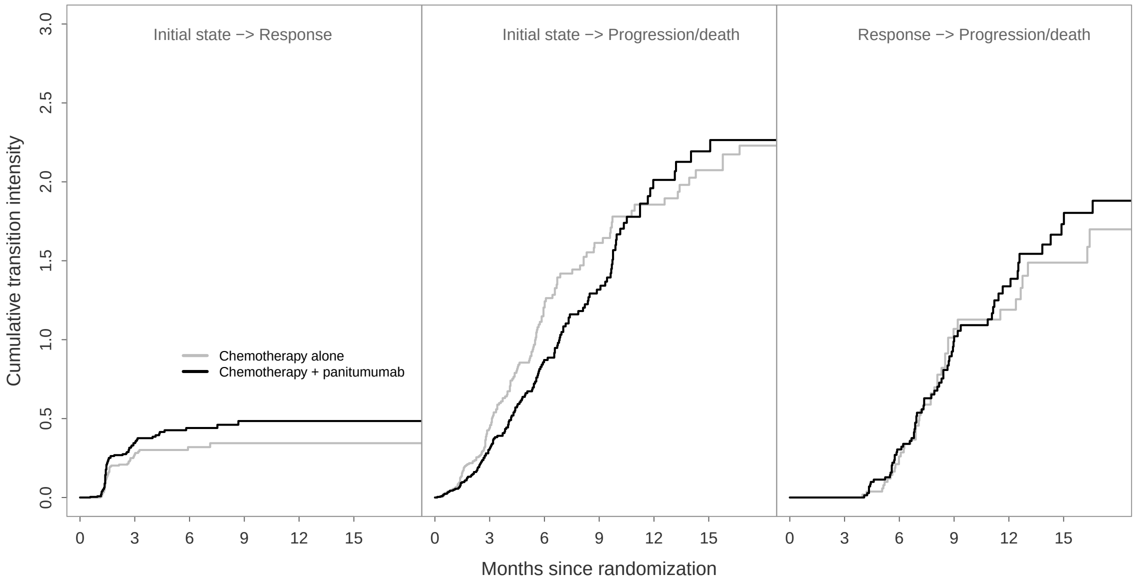

Figure 13. SPECTRUM trial analysis: Nonparametric estimates of the cumulative transition intensities by treatment arm.

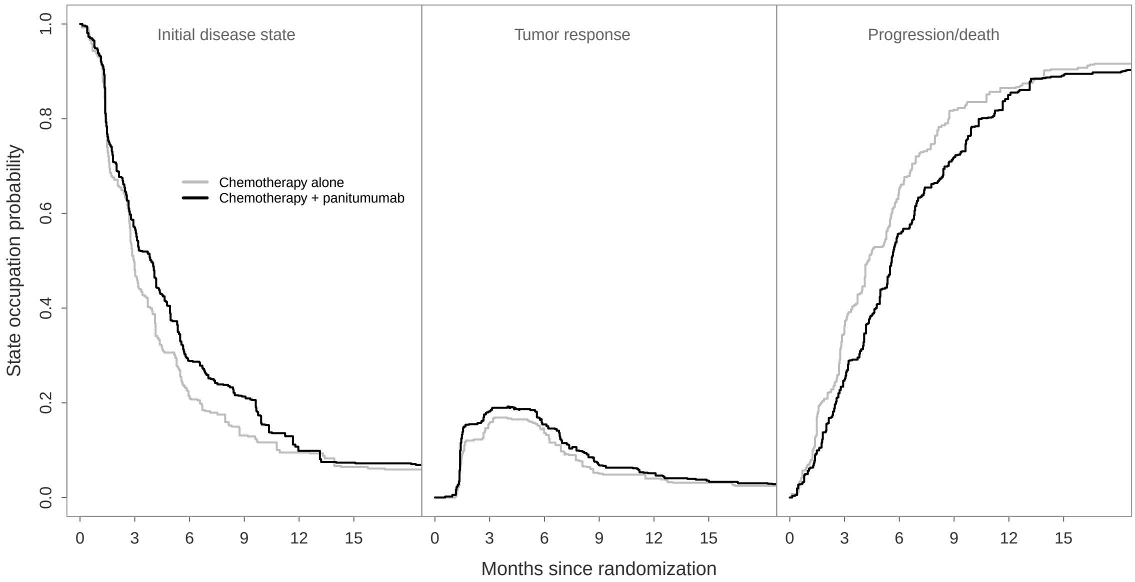

Figure 14. SPECTRUM trial analysis: Nonparametric estimates of the state occupation probabilities by treatment arm.

Table 1

Simulation study: Performance of the proposed inference methods for the true value of the estimated ITR $\mathcal{V}_w(\hat{d}_{n,w})$ for the progression-free survival time (i.e., $w = (1, 1, 0)'$), under a linear optimal decision function f_w^* (scenarios 1 and 2). (Cens: right censoring rate; n: training sample size; MCSD: Monte Carlo standard deviation of the estimates; ASE: Average of the standard error estimates; CP: empirical coverage probability of the 95% confidence interval)

			$\hat{\mathcal{V}}_{n,w}(\hat{d}_{n,w})$				$\hat{\mathcal{V}}^{jk}_{n,w}(\hat{d}_{n,w})$			
Scenario	Cens	n	% error	MCSD	ASE	CP	% error	MCSD	ASE	CP
1	28%	100	3.453	0.304	0.272	0.945	-2.439	0.368	0.272	0.893
		200	2.068	0.208	0.199	0.942	-0.779	0.218	0.199	0.935
		400	1.327	0.140	0.142	0.956	-0.502	0.147	0.142	0.940
	43%	100	4.397	0.318	0.300	0.956	-2.285	0.383	0.300	0.902
		200	3.009	0.221	0.218	0.952	-0.393	0.244	0.218	0.931
		400	1.627	0.151	0.155	0.959	-0.267	0.156	0.155	0.955
	60%	100	5.171	0.382	0.363	0.946	-4.364	0.476	0.363	0.886
		200	3.220	0.264	0.262	0.954	-1.236	0.315	0.262	0.914
		400	1.627	0.181	0.187	0.954	-0.856	0.200	0.187	0.939
2	28%	100	2.023	0.324	0.289	0.953	-0.910	0.331	0.289	0.941
		200	1.407	0.227	0.207	0.949	-0.270	0.228	0.207	0.938
		400	0.625	0.161	0.147	0.950	-0.249	0.162	0.147	0.944
	42%	100	2.472	0.344	0.319	0.948	-0.968	0.351	0.319	0.931
		200	1.839	0.236	0.227	0.947	-0.115	0.242	0.227	0.941
		400	0.901	0.171	0.161	0.941	-0.195	0.174	0.161	0.936
	57%	100	1.892	0.402	0.383	0.948	-3.099	0.438	0.383	0.919
		200	2.002	0.261	0.274	0.962	-0.678	0.272	0.274	0.944
		400	0.763	0.184	0.192	0.970	-0.741	0.188	0.192	0.954

Table 2

Simulation study: Performance of the proposed inference methods for the true value of the estimated individualized treatment rule $\mathcal{V}_w(\hat{d}_{n,w})$ for the progression-free survival time (i.e., $w = (1, 1, 0)'$), under a nonlinear optimal decision function f_w^* (scenarios 3 and 4). (Cens: right censoring rate; n: training sample size; MCSD: Monte Carlo standard deviation of the estimates; ASE: Average of the standard error estimates; CP: empirical coverage probability of the 95% confidence interval)

			$\hat{\mathcal{V}}_{n,w}(\hat{d}_{n,w})$				$\hat{\mathcal{V}}^{jk}_{n,w}(\hat{d}_{n,w})$			
Scenario	Cens	n	% error	MCSD	ASE	CP	% error	MCSD	ASE	CP
3	29%	100	3.511	0.313	0.271	0.958	-1.521	0.361	0.271	0.908
		200	1.992	0.214	0.198	0.959	-0.967	0.234	0.198	0.937
		400	1.012	0.152	0.142	0.957	-0.573	0.156	0.142	0.942
	43%	100	4.064	0.344	0.298	0.953	-2.430	0.401	0.298	0.905
		200	2.574	0.239	0.217	0.938	-0.673	0.266	0.217	0.914
		400	1.379	0.156	0.155	0.957	-0.461	0.162	0.155	0.947
	60%	100	5.864	0.389	0.362	0.947	-3.478	0.466	0.362	0.894
		200	3.404	0.272	0.261	0.962	-0.944	0.316	0.261	0.930
		400	1.826	0.179	0.186	0.961	-0.638	0.197	0.186	0.954
4	29%	100	3.767	0.323	0.271	0.930	-1.907	0.380	0.271	0.887
		200	2.016	0.222	0.198	0.941	-0.895	0.240	0.198	0.920
		400	1.419	0.146	0.142	0.953	-0.107	0.150	0.142	0.946
	43%	100	4.548	0.321	0.298	0.951	-1.714	0.389	0.298	0.901
		200	2.319	0.232	0.216	0.954	-0.840	0.257	0.216	0.925
		400	1.475	0.159	0.155	0.956	-0.184	0.161	0.155	0.945
	59%	100	5.520	0.408	0.364	0.947	-3.631	0.494	0.364	0.883
		200	3.142	0.270	0.262	0.958	-0.975	0.295	0.262	0.933
		400	2.067	0.189	0.187	0.960	-0.180	0.197	0.187	0.949
