# Supplementary material for: Estimating optimal individualized treatment rules with multistate processes
Source: Biometrics. Author manuscript; Available in PMC 2024 Feb 16. (PMC10553793; doi:10.1111/biom.13864)
Supplement: R code description [file NIHMS1931646-supplement-R_code_description.docx]

**Instructions for the code msowl.R**

**Description**

This code provides functions for the estimation of optimal individualized treatment rules with multistate processes via outcome weighted learning. These functions also provide the estimated value function of an individualized treatment rule as well as its (estimated) standard error.

**Dependencies**

Package kernlab.

**Main functions**

i) itr: Estimates an optimal individualized treatment rule with multistate processes.

ii) V_d: Computes the estimator the value function of a treatment rule and its standard error.

**Inputs for itr**

data: A data frame with multiple records per individual containing the variables:

id: The unique id for the individuals

t1: Starting time of the interval

t2: Stopping time of the interval

s1: The state at t1

s2: The state visited at t2

feat: a specification of the covariates/features *Z* to be used for tailoring treatment to individuals. feat is a vector containing the variable names for the latter variables

w: the weight of patient preferences that satisfies 0 <= min(w) < max(w) <= 1. length(w) should be equal to the number of states of the multistate process of interest.

tau: Maximum time to be considered

kernel: a specification of the form of the decision function. kernel can be set to ‘linear’ or ‘rbf’ (radian basis function Gaussian kernel)

sigma: the parameter σ of the Gaussian kernel if kernel = `rbf’

lambda: the penalty parameter λ

SE: logical value: if TRUE, the function returns the estimated standard error of the estimated value function of the estimated optimal individualized treatment rule

**Inputs for V_d**

data: A data frame with multiple records per individual containing the variables:

id: The unique id for the individuals

t1: Starting time of the interval

t2: Stopping time of the interval

s1: The state at t1

s2: The state visited at t2

feat: a specification of the covariates/features *Z* to be used for tailoring treatment to individuals. feat is a vector containing the variable names for the latter variables

w: the weight of patient preferences that satisfies 0 < min(w) < max(w) < 1. length(w) should be equal to the number of states of the multistate process of interest.

tau: Maximum time to be considered

kernel: a specification of the form of the decision function. kernel can be set to ‘linear’ or ‘rbf’ (radian basis function Gaussian kernel)

sigma: the parameter σ of the Gaussian kernel if kernel = `rbf’

lambda: the penalty parameter λ

SE: logical value: if TRUE, the function returns the estimated standard error of the estimated value function of the estimated optimal individualized treatment rule

**Examples**

setwd("C:/mydir")

source("msowl.R")

library(foreign)

data <- read.csv("example_data.csv")

The dataset “example_data.csv” contains observations from the multistate process of the form:

Estimate an optimal individual treatment rule for prolonging the time spent in State 2 based on a linear decision function and consider the process up to time *τ* = 3:

fit <- itr(data=data, feat=c("Z1", "Z2"), w = c(0, 1, 0), tau = 3, lambda=1, kernel=`linear’, SE=TRUE)

#Estimates of the coefficients of the optimal linear decision function

fit$beta_opt

#Estimated value function of the estimated rule

fit$V_opt

#Estimated standard error of the value function of the estimated rule

fit$se_V_opt

Estimate the value function and of the latter estimated optimal treatment rule and its standard error using the function V_d:

V_d(data=data, w=c(0, 1, 0), tau=3, dec.fun=fit$fit, feat=c("Z1", "Z2"), SE = TRUE)

Estimate the value function and of the fixed rule that assigns treatment 1 to everyone, along with its standard error using the function:

V_d(data=data, w=c(0, 1, 0), tau=3, dec.fun=1, feat=c("Z1", "Z2"), SE = TRUE)

Estimate the value function and of the fixed rule that assigns treatment -1 to everyone, along with its standard error using the function:

V_d(data=data, w=c(0, 1, 0), tau=3, dec.fun=-1, feat=c("Z1", "Z2"), SE = TRUE)
